# Will L‐PRF Be the Future of Endodontic Microsurgery? A Series of Case Reports

**DOI:** 10.1002/cre2.70198

**Published:** 2025-12-15

**Authors:** Natrah Ahmad Fuad, Panagiotis Pitros, Graeme Brown, Eleni Besi

**Affiliations:** ^1^ Department of Restorative Dentistry, Faculty of Dentistry University of Malaya Kuala Lumpur Malaysia; ^2^ Department of Restorative Dentistry Edinburgh Dental Institute Edinburgh UK; ^3^ Department of Oral Surgery Edinburgh Dental Institute Edinburgh UK; ^4^ Private Practice limited to Oral Surgery Cyprus Greece; ^5^ Specialty Registrar in Orthodontics (StR), Manchester Dental Hospital Manchester UK; ^6^ Barts and The London School of Medicine and Dentistry London UK

**Keywords:** endodontic, L‐PRF, microsurgery, platelet‐rich fibrin, quality of life

## Abstract

**Objectives:**

This case series aimed to evaluate the healing potential of apical tissues with large periapical radiolucencies (> 10 mm) after apical microsurgery with L‐PRF. The secondary objectives were to evaluate L‐PRF's benefits and adverse effects as well as to aid in the development of a clinical protocol.

**Materials and Methods:**

This case series was conducted in accordance with the Preferred Reporting Items for Case Reports in Endodontics (PRICE) 2020 guidelines. Thirteen patients with persistent endodontic infections, unresponsive to nonsurgical root canal treatment/retreatment, were treated at the Restorative and Oral Surgery Departments with endodontic microsurgery. L‐PRF preparation followed Choukroun et al. (2001) and the L‐PRF 2018 guidelines under the supervision of an experienced consultant. Postoperative follow‐up included a phone call at 24 h to assess pain, swelling, and daily functions. Sutures were removed at 7 days, and a 6‐month clinical and radiographic review was conducted. The clinical assessment included patient‐reported symptoms and extraoral and intraoral examinations. Periapical radiographs were assessed for periapical healing based on Rud et al.'s (1972) radiographic criteria. Radiographs were reviewed by one clinician under standardized conditions.

**Results:**

Histopathological analyses identified 76.9% (*n* = 10) radicular cysts and 23.0% (*n* = 3) periapical granulomas from the 13 cases. At the 6‐month review, 76.9% (*n* = 10) showed incomplete healing, 15.4% (*n* = 2) demonstrated complete healing, and 7.7% (*n* = 1) had incomplete healing at 4 months. All patients remained asymptomatic with no reported complaints. Radiographic assessments showed a significant reduction in the size of periapical radiolucency in all cases. At 24 h, 69.2% (*n* = 9) reported no pain, while mild pain was noted in 15.4% (*n* = 2). Swelling was observed in 69.2% (*n* = 9) and absent in 15.4% (*n* = 2), with missing records for 15.4% (*n* = 2).

**Conclusion:**

L‐PRF appears beneficial in endodontic microsurgery. However, larger, low‐bias studies with extended follow‐up periods are needed for definitive conclusions on its application.

## 1 Introduction

Endodontic microsurgery is a treatment option for cases where conventional root canal therapy has failed. With the increasing preference for tooth preservation over extraction, patients are more likely to consent to apical surgery (Azarpazhooh et al. 2013). According to the 2020 Periradicular Surgery Guidelines by the Royal College of Surgeons, apical surgery is indicated when symptoms persist or periradicular disease progresses despite a satisfactory nonsurgical root canal treatment. However, large periapical lesions (> 5 mm) are associated with reduced healing rates and consequently prolonged healing times following apical surgery (Wang et al. 2004; von Arx et al. 2010). A long‐standing debate remains on whether to introduce grafting materials during apical surgery for large lesions or to allow natural healing. The histological outcome of wound healing in these cases can be classified as either repair or regeneration, depending on factors such as the nature of the periapical wound, the availability of growth factors and stem cells, and the microenvironmental cues of the surgical site (Lin et al. 2010). Regeneration is the preferred outcome, as it restores the original architecture and biological function of the tissue, but it does not always occur.

Advancements in microsurgical techniques have introduced regenerative strategies to optimize healing outcomes in endodontic microsurgery. These approaches include the use of barrier membranes and platelet concentrates, with or without concurrent bone grafting, to promote healing. While regenerative techniques have demonstrated high success rates in implant and periodontal surgery (Bashutski and Wang 2009), evidence regarding their effectiveness in endodontic microsurgery remains inconclusive. Some studies have supported their use (Sanchez‐Torres et al. 2014; Deng et al. 2016), whereas others have not (von Arx et al. 2011; Corbella et al. 2016). The goal of regenerative techniques in apical surgery is to create a favorable microenvironment for tissue and bone regeneration in periapical defects (Bashutski and Wang 2009), thereby accelerating wound healing.

One such regenerative material is platelet‐rich fibrin (PRF), which has shown promise in promoting rapid wound healing and bone regeneration in apical surgeries, though most supporting studies are case reports (Singh et al. 2013; Angerame et al. 2015; Pawar et al. 2015; Garg et al. 2023; You et al. 2023). While PRF's benefits remain largely empirical, You et al. (2023) reported significantly greater bone deposition at follow‐up when PRF was applied to the surgical site compared to cases without PRF. PRF is an autologous material with a simple, single‐step preparation that does not require external additives, reducing the risk of immune rejection. An observational study in Edinburgh further supports its use, demonstrating positive outcomes in the prevention of medication‐related osteonecrosis of the jaw (MRONJ) in 39 patients receiving anti‐resorptive and antiangiogenic medications following tooth extractions with L‐PRF application (Besi and Pitros 2024). Additionally, PRF has been associated with improved postoperative quality of life (QoL) in endodontic surgeries (Del Fabbro 2012; Angerame et al. 2015).

Various regenerative materials have been investigated in endodontic microsurgery, including collagen membranes (Parmar et al. 2019), bovine‐derived hydroxyapatite (Dominiak et al. 2009), expanded polytetrafluoroethylene (e‐PTFE) membranes (Pecora et al. 1995), calcium sulfate (Pecora et al. 2001; Taschieri et al. 2007; Wang et al. 2017), and autologous platelet concentrates such as platelet‐rich plasma (PRP) and leukocyte‐ and platelet‐rich fibrin (L‐PRF) (Dominiak et al. 2009; Dhiman et al. 2015; Meschi et al. 2020). A recent systematic review and meta‐analysis reported that the use of regenerative materials in endodontic microsurgery significantly improved healing outcomes compared to conventional approaches (RR: 0.42; 95% CI: 0.26–0.68; *p* < 0.001) (Liu et al. 2021). It is not within the scope of this case series to compare the different regenerative materials. This case series shows the benefits of L‐PRF membranes as adjuncts for apical surgery in 13 cases that underwent apical microsurgery. L‐PRF is the second generation of platelet concentrates, which is of autologous source and does not require the addition of extrinsic factors or anti‐coagulants for activation (Chuokroun et al. 2001).

The primary aim of this case series was to evaluate the healing potential of apical tissues with large periapical radiolucencies (> 10 mm) after apical microsurgery with the addition of L‐PRF. The secondary objectives were: (i) to evaluate the beneficial effects of L‐PRF in patients, (ii) to evaluate for any adverse effects associated with the use of L‐PRF, and (iii) to construct a protocol for appropriate cases.

## 2 Report

### 2.1 Materials and Methods

Thirteen patients (8 males and 5 females) within the age range of 17–68 years were included in this case series (Table 1). Informed, valid consent was obtained for clinical examination and further investigation from each patient. This case series has been prepared according to the Preferred Reporting Items for Case Reports in Endodontics (PRICE) 2020 guidelines (Nagendrababu et al. 2020) (Appendix 3). Approval was obtained from the Quality Improvement Project Assessment Team (QIPAT), NHS Lothian. All patients required apical surgery after satisfactory root canal treatment/retreatment (henceforth root canal treatment) due to persistent signs of infection and inflammation. Persistent signs of infection or inflammation include a persistent sinus tract, non‐resolving periapical radiolucency, and/or ongoing symptomatic teeth. All patients had apical radiolucencies measuring 10 mm or more (Table 1). The evaluation of the apical tissues was made based on periapical radiographs with or without the addition of cone‐beam CT (CBCT). All cases were treated at the Restorative and Oral Surgery Departments at the Edinburgh Dental Institute by multiple clinicians. The root canal treatment was performed at the Restorative Department, whereas the apical surgery with L‐PRF was performed at the Oral Surgery Department under the supervision of a consultant who is familiar with the L‐PRF protocol. Eleven cases involved the anterior maxillary region and two cases involved the anterior mandibular region (Table 1). The root canal treatment was performed as per the protocol in the Restorative Department (Appendix 1).

**Table 1:** Details of the 13 cases included in this case series.

Case	Age	Sex	Medical history	Smoking status	Area of lesion	Dimensions of lesion	Number of L‐PRF membranes/plug	Histopathology diagnosis
1	38	Male	Fit and well No known drug allergies	Never smoker	Anterior maxilla involving the upper right lateral incisor (tooth 12)	21 mm × 18 mm × 18 mm	5 membranes	Radicular cyst
2	68	Male	Atrial fibrillation Hypertension Type II diabetes mellitus No known drug allergies	Ex‐smoker (20 years cessation)	Anterior maxilla involving the upper left central incisor (tooth 21) and lateral incisor (tooth 22)	17 mm × 19 mm × 15 mm	4 membranes	Radicular cyst
3	36	Female	Fit and well No known drug allergies	Never smoker	Anterior maxilla involving the upper right central incisor (tooth 11) and lateral incisor (tooth 12)	14 mm × 14 mm × 14 mm	4 membranes	Radicular cyst
4	36	Female	Fit and well No known allergies	Never smoker	Anterior maxilla involving the upper left central incisor (tooth 21)	12 mm × 14 mm × 10 mm	No record	Radicular cyst
5	62	Female	Hypertension No known drug allergies	Never smoker	Anterior maxilla involving the upper left central incisor (tooth 21)	18 mm × 18 mm × 18 mm	3 membranes	Periapical granuloma
6	49	Male	Asthma No known drug allergies	Never smoker	Anterior maxilla involving the upper right central incisor (tooth 11) and lateral incisor (tooth 12)	14.5 mm × 20 mm × 15.5 mm	4 membranes	Radicular cyst
7	25	Male	Fit and well No known drug allergies	Vapes	Anterior maxilla involving the upper right lateral incisor (tooth 12)	17.5 mm × 15.5 mm × 17 mm	3 membranes, 1 plug	Radicular cyst
8	43	Male	Fit and well No known drug allergies	Never smoker	Anterior maxilla involving the upper right canine (tooth 13)	19 mm × 21 mm × 18 mm	4 membranes	Periapical granuloma
9	28	Male	Fit and well No known drug allergies	Never smoker	Anterior maxilla involving the upper right lateral incisor (tooth 12)	16.5 mm × 16.5 mm × 16.5 mm	No record	Periapical granuloma
10	34	Male	Slipped disk On gabapentin No known drug allergies	Never smoker	Anterior maxilla involving the upper left lateral incisor (tooth 22)	11.5 mm × 12.5 mm × 11 mm	No record	Radicular cyst
11	17	Male	Fit and well No known drug allergies	Never smoker	Anterior maxilla involving the upper left central incisor (tooth 11)	10 mm × 15 mm × 2 mm	No record	Radicular cyst
12	37	Female	Fit and well No known drug allergies	Smoker—5 cigarettes/week	Lower anterior mandible associated with lower right, left central, and lateral incisors (teeth 31, 32, 41, and 42)	23 mm × 10 mm × 10.5 mm	No record	Radicular cyst
13	60	Female	Arthritis Allergic to pork	Never smoker	Anterior maxilla involving the upper right central incisor (tooth 11)	15 mm × 5 mm × 5 mm	4 membranes	Radicular cyst

### 2.2 Endodontic Microsurgery Procedure

**Figure 1:**
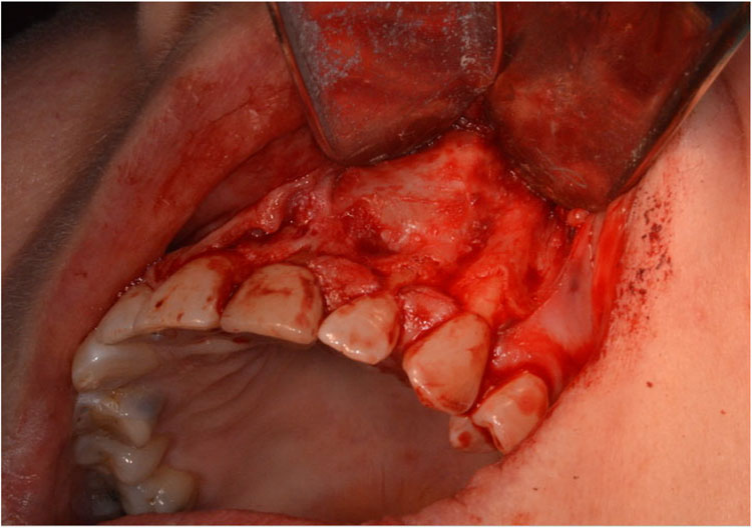
Representative case (Case 5) showing papilla base incisions and full thickness flap reflection.

**Figure 2:**
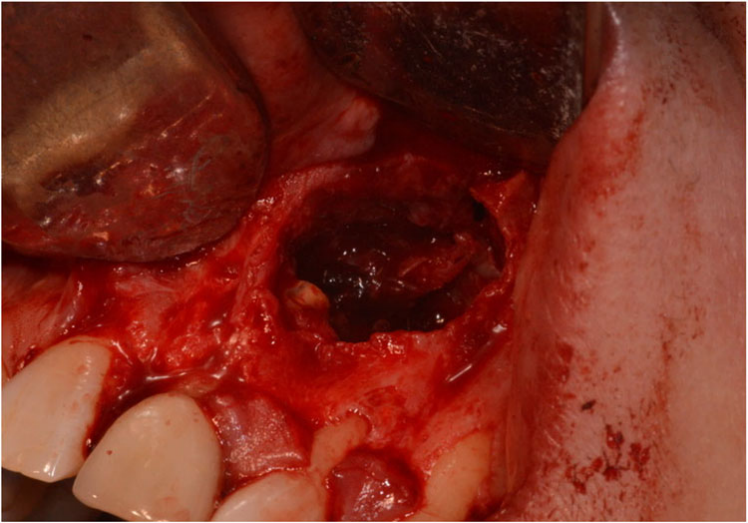
Representative case (Case 5) showing the osteotomy site and after the enucleation of granulation tissue.

**Figure 3:**
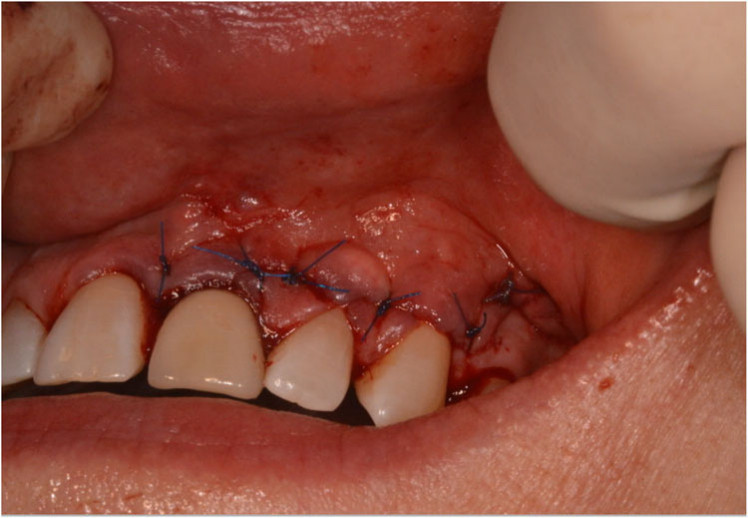
Representative case (Case 5) showing flap repositioned and sutures in place.

The endodontic microsurgery procedures were performed by multiple clinicians, either an endodontic postgraduate trainee, an oral surgery trainee, or a specialist. A standardized protocol was adopted, and an operating microscope was used (Global G6 series dental microscope, Global Surgical Corporation, or Carl Zeiss Extaro 300, Nuview Ltd, Vine House, North Woodchester, Gloucestershire). After the administration of local anesthetic, 2% Lidocaine (Septodont Ltd. Lignospan Special, 20 mg/mL + 12.5 µg/mL) infiltration, incisions were made based on the extension that is required for adequate visualization of the apical lesion. Papilla base incisions were made, joined by intrasulcular incisions (Velvart 2002). Either a triangular or two‐sided vertical releasing incision was made, which depended on the amount of visualization required. After a full thickness flap was raised (Figure 1), osteotomy was made starting at the bone fenestration, if present, or at an estimated position of the apical lesion based on the periapical radiograph or cone beam CT (CBCT). This was then followed by the removal of granulation tissue (all apical tissues removed will be referred to as granulation tissue before histopathological evaluation) (Figure 2). Once the granulation tissue has been removed, the bony cavity was inspected for any perforations of the buccal and/or palatal cortical plates as well as the nasal floor. This was then followed by 3 mm of root‐end resection, root‐end preparation, and root‐end retro‐filling of the root‐treated tooth. Calcium silicate material (Well‐Root PT Bioceramic Putty, Well‐Root ST, Vericom) or Mineral Trioxide Aggregate (MTA) (MTA Angelus, Angelus Industria de Produtos Odontologicos S/A, Brazil) was used as the root‐end filling material. The bony cavity was then ready to receive the L‐PRF membrane. Once the L‐PRF membranes were placed satisfactorily and bleeding was controlled, the surgical site was sutured with non‐resorbable sutures 5/0 or 6/0, either PTFE (Coreflon Silver, PTFE monofil nonabsorbable surgical suture, Implacore Sp. Zo.o.o.ul. Polska 94a 60‐401 Ponań, Poland) or Prolene (Ethicon, Johnson & Johnson Medical NV, Belgium) sutures (Figure 3). The granulation tissue removed from the bony crypt was sent for histopathological analysis. Postoperative instructions were then given to the patients in terms of complications, management of the complications, diet, oral hygiene practice, as well as suture removal appointment. A postoperative periapical radiograph was taken right after the completion of the surgery. Suture removal was done 7 days later.

### 2.3 Protocol for the Preparation of L‐PRF

**Figure 4:**
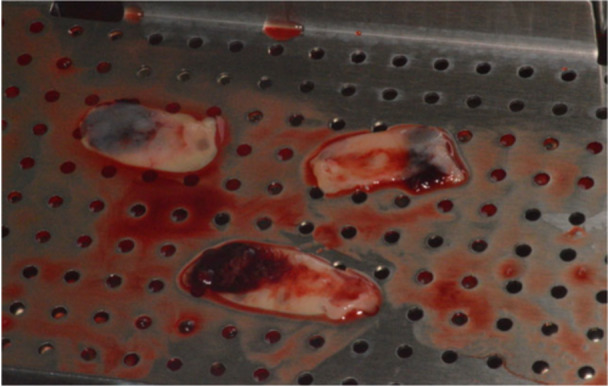
L‐PRF membranes on the Xpression box.

L‐PRF membrane is an analogous membrane that is obtained from each individual's own blood. Venous blood was taken from each patient at the median cubital fossa. The amount of blood required depended on the number of L‐PRF membranes needed, which coincided with the lesion size. Generally, about 3–4 tubes of blood will be required (9 mL tubes). The tubes with the collected whole blood were then placed in a centrifuge machine (IntraSpin System, Intra‐Lock International Inc.) opposite to each other to ensure the centrifuge machine is balanced, for 12 min at RPM 2700. After 12 min, L‐PRF clot was produced which consisted of the platelet‐poor plasma (PPP) (first layer), PRP or the fibrin clot/L‐PRF (second layer), and the red blood cell (third layer). The red blood cell was of no use and was removed. The L‐PRF clots were then placed in an Xpression box (Xpression Intra‐Lock International Inc.) for 5 min, which compressed the L‐PRF clots by gravity to express out the serum from the fibrin clot forming L‐PRF membranes with a consistent thickness of 1 mm (Figure 4). The L‐PRF membranes were viable for 2.5–3 h as long as they were rehydrated with exudate (Pinto et al. 2017). After this step, the L‐PRF membranes produced were placed into the defect at the desired position, which most of the time involved the palatal/lingual wall and/or adjacent to the nasal floor and the labial/buccal wall. The L‐PRF protocol diagram is shown in Appendix 2.

### 2.4 Follow‐up Regime

All patients received a follow‐up phone call 24 h after surgery to assess their recovery. They were asked about pain severity, swelling, and their ability to perform daily activities, which were described in their own words. Sutures were removed 7 days post‐surgery, followed by a review appointment 6 months later, which included both clinical and radiographic evaluations. The clinical review at 6 months included: (i) assessing the patient's complaints, (ii) performing an extraoral examination, with a focus on evaluating the soft tissues, particularly their appearance when smiling, and (iii) performing an intraoral examination, which involved assessing the existing restorations, evaluating soft tissues particularly for scar formation and recession, conducting percussion and palpation tests, and assessing tooth mobility. The follow‐up radiographs taken were periapical radiographs, to assess for periapical healing, based on the radiographic criteria for the assessment of healing after endodontic microsurgery (Rud et al. 1972) (Table 2). The radiographs were viewed by only one clinician (N.A.F.) in a darkened room, viewed on one NHS Lothian computer monitor.

**Table 2:** Rud et al. (1972) radiographic criteria.

Radiographic criteria	Description
Complete healing	Reformation of PDL space. The width of the PDL space in the apical region may be widened up to twice the normal width around a non‐involved part of the root. Healing with hard tissue evident.
Incomplete healing (scar tissue)	A rarefaction exists compared to a postoperative or a previous follow‐up radiograph, either a decrease in size or a stationarity. The periphery is irregular, and the rarefaction is often located asymmetrically around the root apex.
Uncertain healing	Some degree of bone regeneration exists, a reduction in the size of the rarefaction compared to the postoperative or previous follow‐up radiograph. The periphery of the rarefaction is nearly always circular or semicircular, and the rarefaction is usually symmetrically placed around the root apex.
Unsatisfactory healing (failure)	The radiographic signs are similar for the uncertain healing, but the rarefaction is either enlarged or unchanged.

## 3 Results

**Figure 5:**
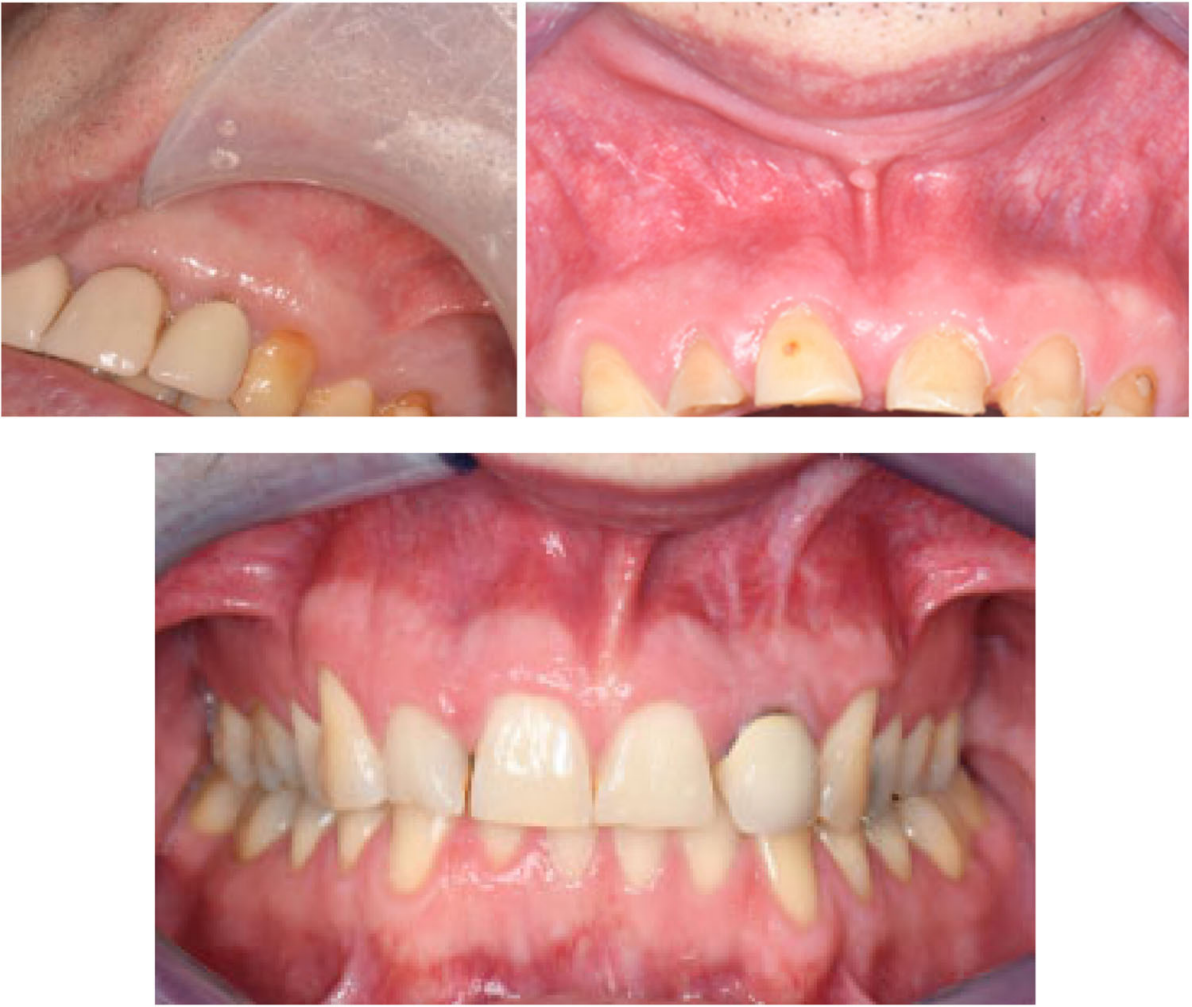
Representative case (Case 2 [left], 6 [right], 10 [bottom]), showing satisfactory soft tissue healing at the 6‐month review.

Of the 13 cases, 76.9% (*n* = 10) demonstrated incomplete healing at the 6‐month review, 15.4% (*n* = 2) achieved complete healing, and 7.7% (*n* = 1) exhibited incomplete healing at the 4‐month review (Table 3). Regarding postoperative pain at 24 h, 69.2% (*n* = 9) reported no pain, 15.4% (*n* = 2) experienced mild pain, and no record was available for the remaining 15.4% (*n* = 2) (Table 4). Similarly, 69.2% (*n* = 9) presented with swelling at 24 h, 15.4% (*n* = 2) had no swelling, and data were missing for the remaining 15.4% (*n* = 2) (Table 4). At the 6‐month review, all patients were asymptomatic and reported satisfaction with the healing outcome. Figure 5A–C illustrates representative examples of healed soft tissue at the 6‐month review. Histopathological analysis revealed that radicular cysts were more prevalent than periapical granulomas, with 76.9% (*n* = 10) diagnosed as radicular cysts and 23.1% (*n* = 3) as periapical granulomas (Table 5).

**Table 3:** Radiographic healing of the cases at 6‐month review.

Case	Periapical healing (as per Rud et al. 1972)
1	Incomplete healing
2	Incomplete healing
3	Incomplete healing
4	Incomplete healing
5	Incomplete healing
6	Incomplete healing
7	Incomplete healing
8	Complete healing
9	Incomplete healing
10	Incomplete healing
11	Complete healing
12	Incomplete healing for teeth 42 and 31. Complete healing for tooth 32
*13	Incomplete healing*

* This review was done at 4 months instead of 6 months

**Table 4:** One‐day postoperative phone assessment on pain and swelling.

Case	Postoperative pain at 24 h	Postoperative swelling at 24 h
1	Mild pain	Yes
2	No	Yes
3	No	Yes
4	No	Yes
5	No record	No record
6	No	Yes
7	No	Yes
8	No	Yes
9	No	No
10	No	Yes
11	Mild	No
12	No record	No record
13	No	Yes

**Table 5:** Histopathological diagnosis of apical pathosis.

Diagnosis	Counts
Odontogenic (radicular) cyst	10 (76.9%)
Periapical granuloma	3 (23.0%)

The clinical evaluation at the 6‐month review (except for case 13, which was reviewed at 4 months) confirmed that all 13 patients remained asymptomatic, with no reported complaints. The surrounding tissues exhibited good healing, all coronal restorations remained intact, and no cases showed increased tooth mobility or noticeable gingival recession and scarring. All teeth were not tender to percussion, and the surrounding tissues were not tender to palpation. Radiographic assessment at 6 months demonstrated a significant reduction in the periapical radiolucency in all cases, attributed to hard tissue deposition observed in the periapical radiographs (Tables 6, 7, 8, 9, 10, 11, 12, 13, 14, 15, 16, 17). Similarly, at the 4‐month review, case 13 showed comparable findings (Table 18). Notably, two cases (15.4%) exhibited complete healing of the apical tissues (Tables 13 and 16).

**Table 6:** Case 1 radiographs in sequence: Tooth 12.

Pre‐op CBCT	Pre‐op IOPA	Post‐op IOPA	Post‐op IOPA (6 months following surgery)
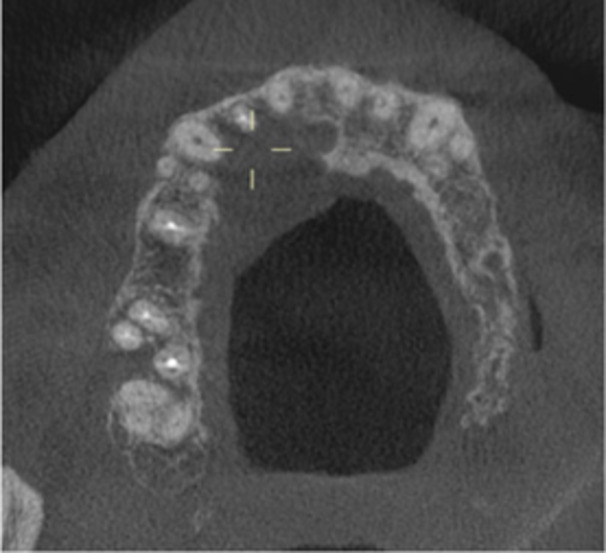 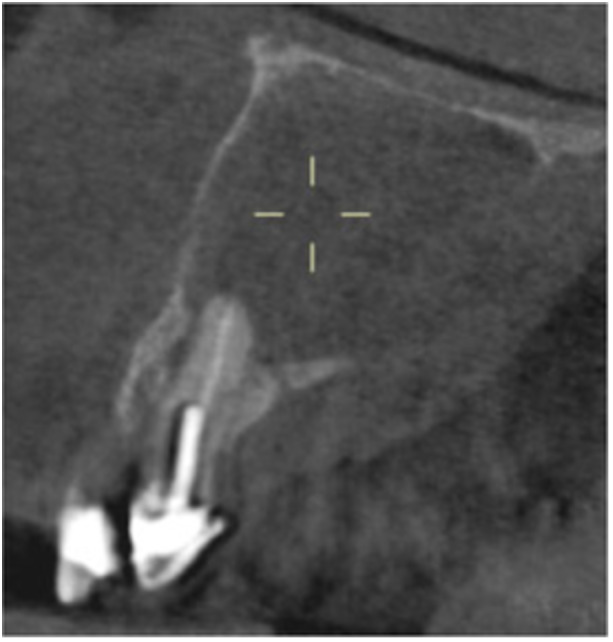	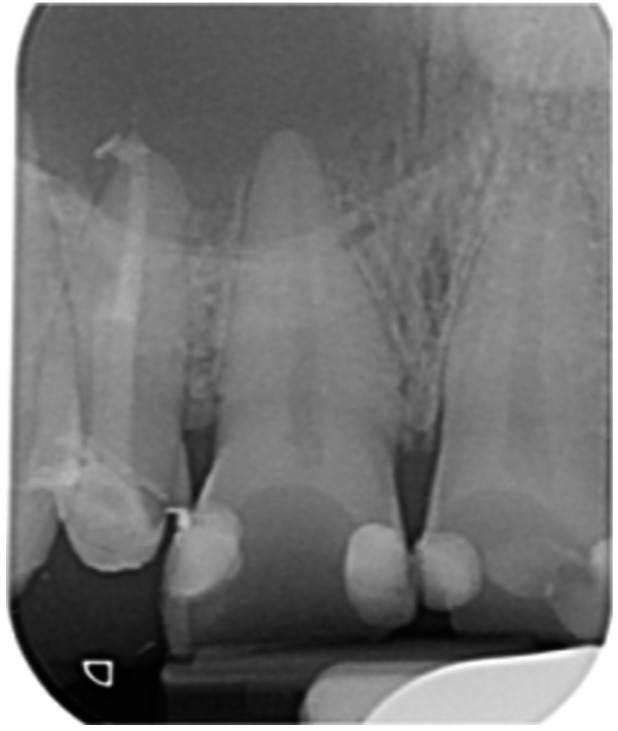	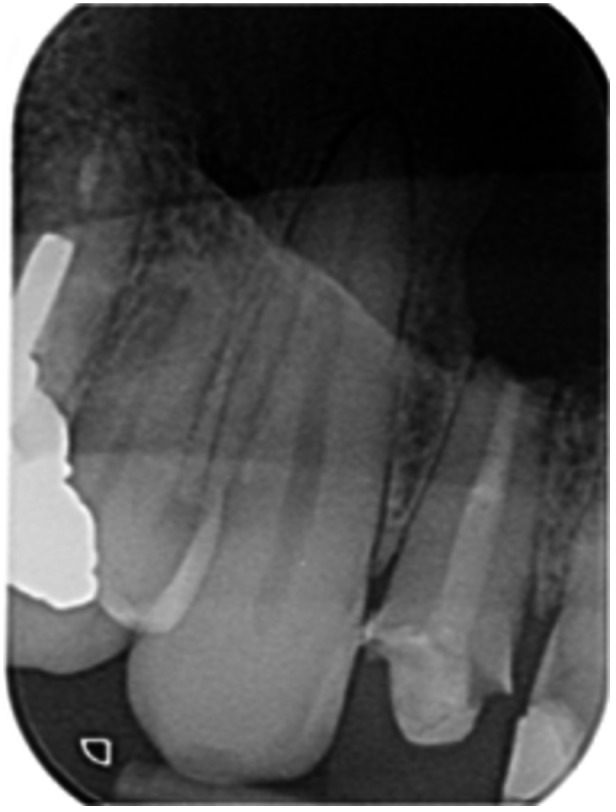	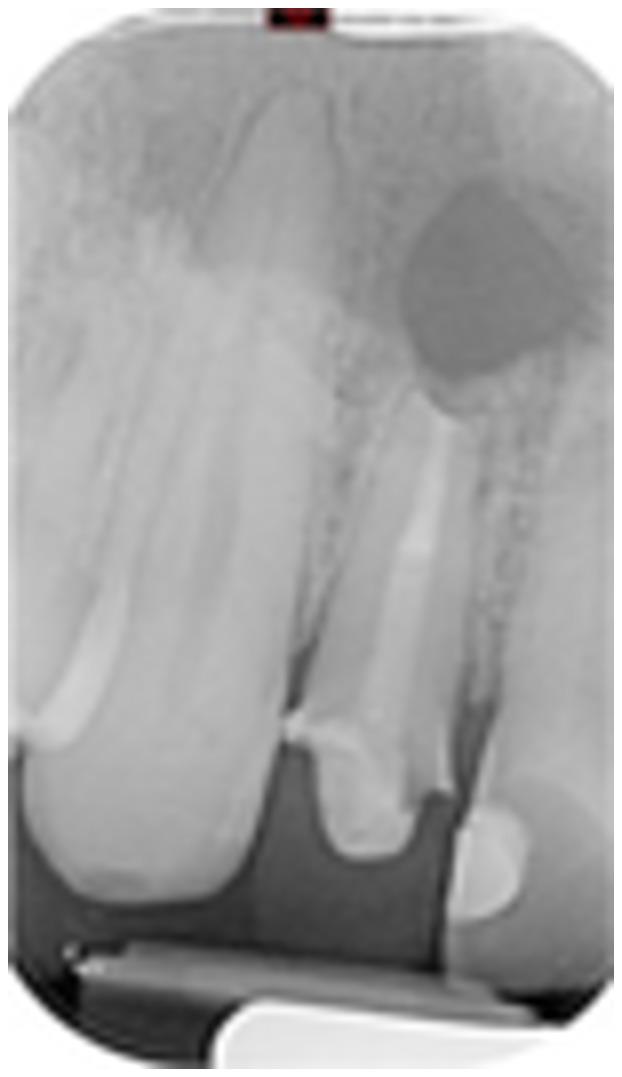

Case 1: The preoperative CBCT and periapical radiograph show an extensive periapical radiolucency extending from teeth 11 to 13. Only tooth 12 contributed to the infection and inflammation; therefore, apical surgery was only performed on tooth 12 following satisfactory nonsurgical root canal treatment. At 6 months, marked healing can be seen, evidenced by a reduction in the periapical radiolucency size, now confined to the apical region of tooth 12 only.

**Table 7:** Case 2 radiographs in sequence: Teeth 21 and 22.

Pre‐op CBCT	Pre‐op IOPA	Post‐op IOPA	Post‐op IOPA (6 months following surgery)
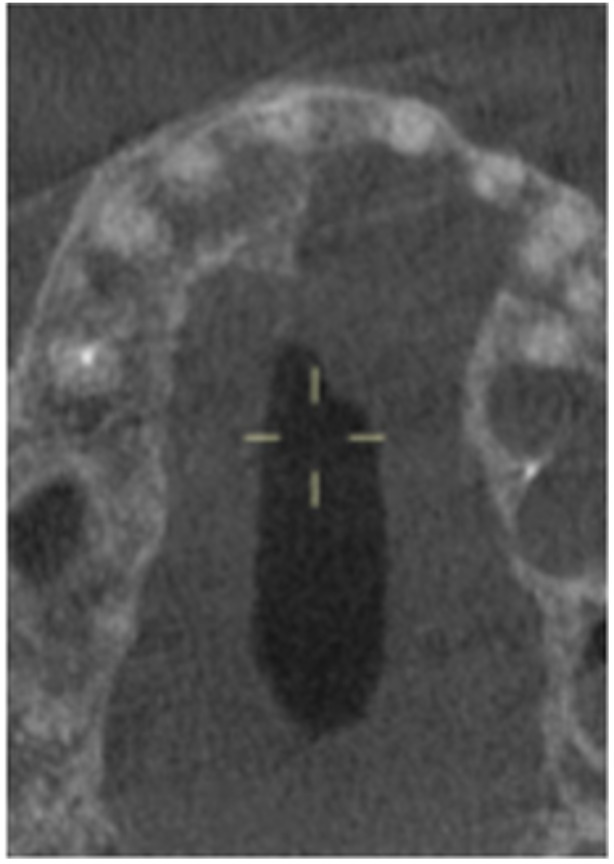 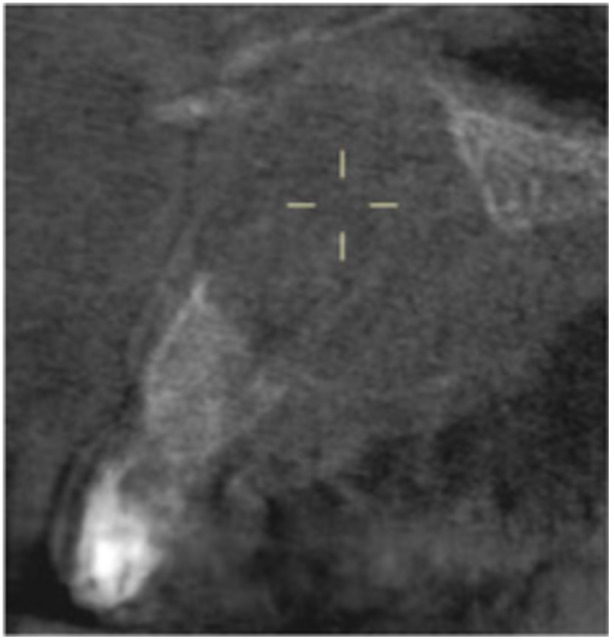	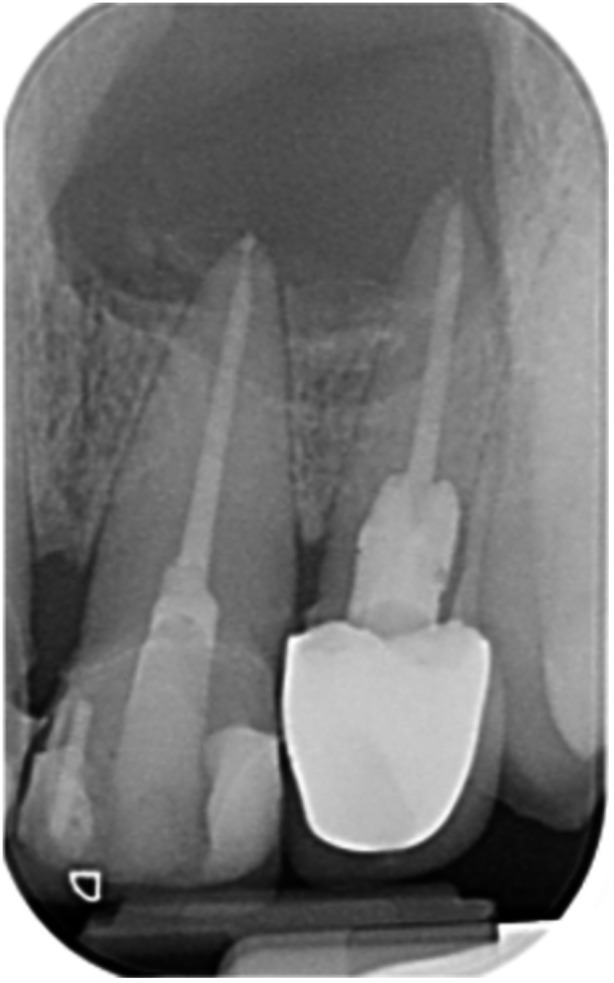	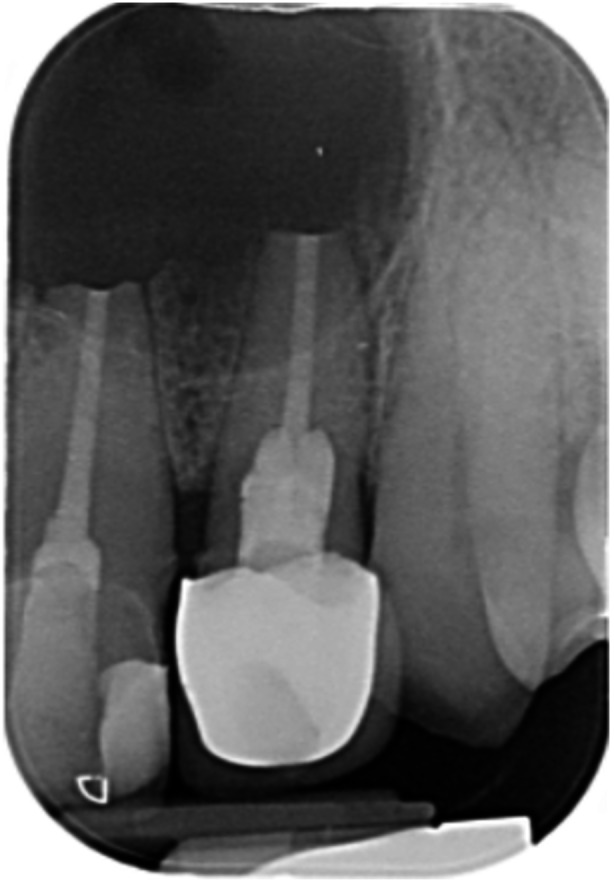	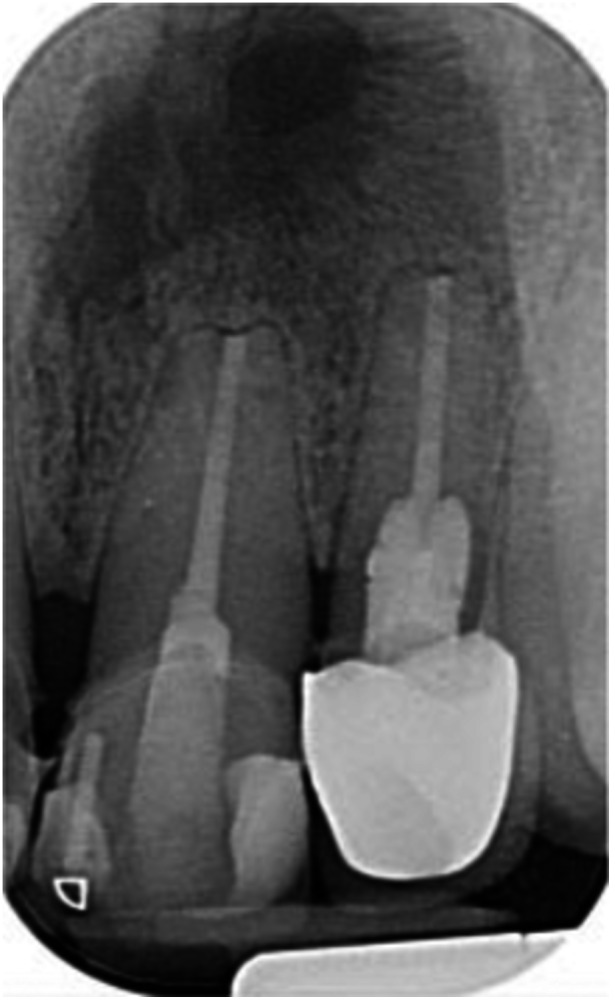

Case 2: The preoperative CBCT and periapical radiograph show an extensive periapical radiolucency involving teeth 21 and 22, extending very close to teeth 11 and 23, with a loss of the palatal cortical bone. Apical surgery was performed on teeth 21 and 22. At 6 months, marked healing can be seen, evidenced by a reduction in the periapical radiolucency size and reattachment of the PDL around the roots of teeth 21 and 22.

**Table 8:** Case 3 radiographs in sequence: Teeth 11 and 12.

Pre‐op CBCT	Pre‐op IOPA	Post‐op IOPA	Post‐op IOPA (6 months following surgery)
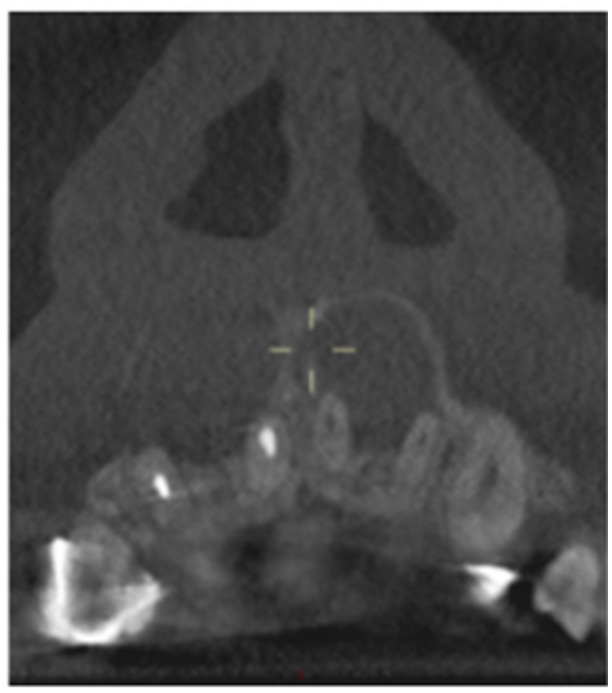 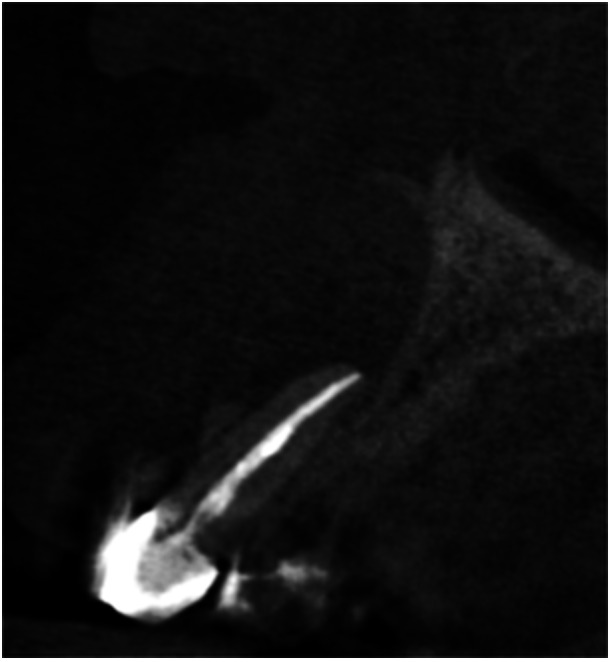	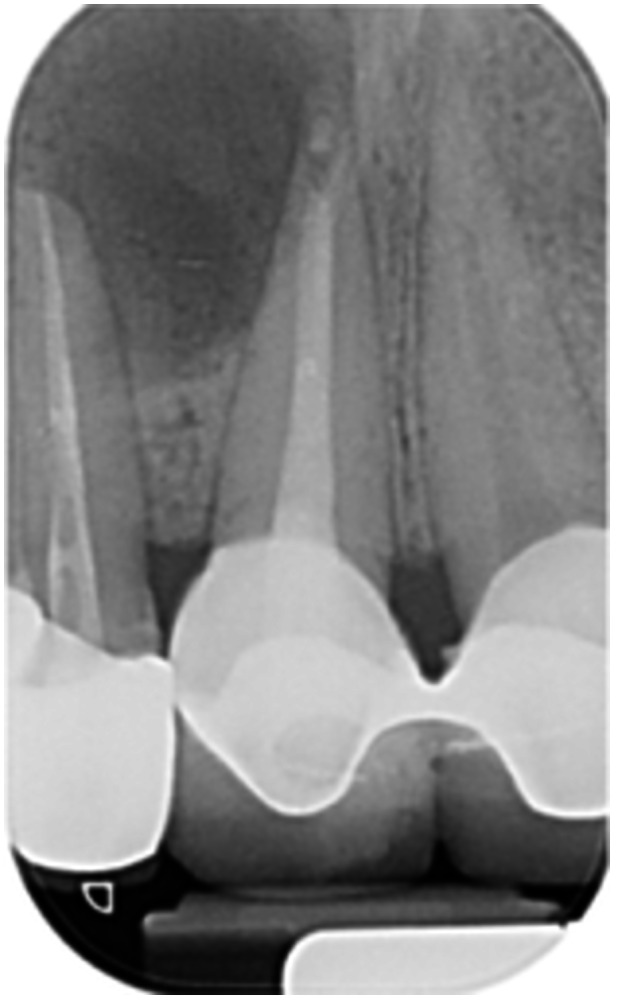	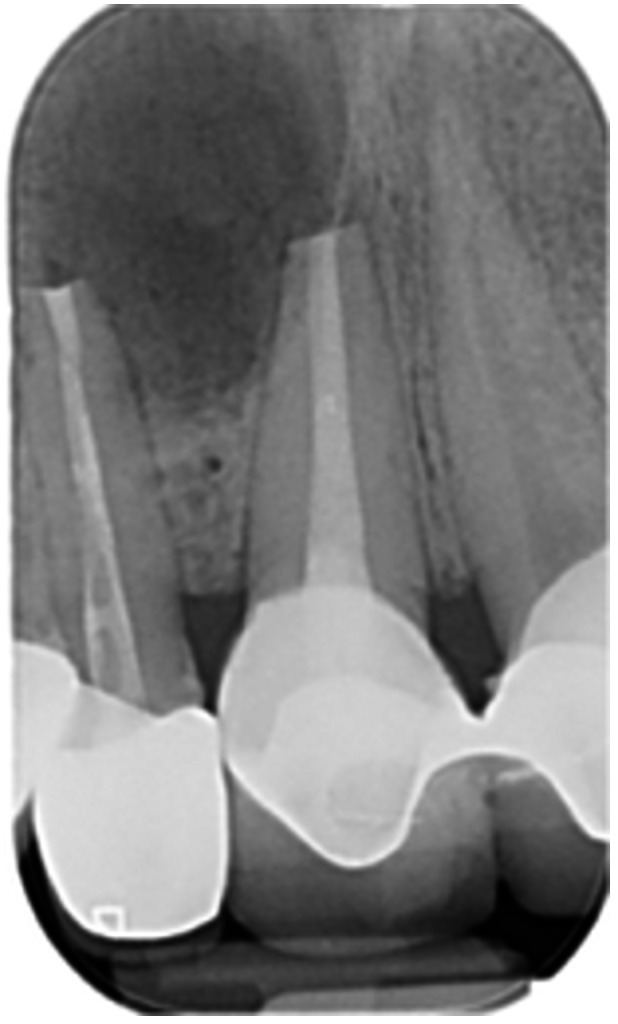	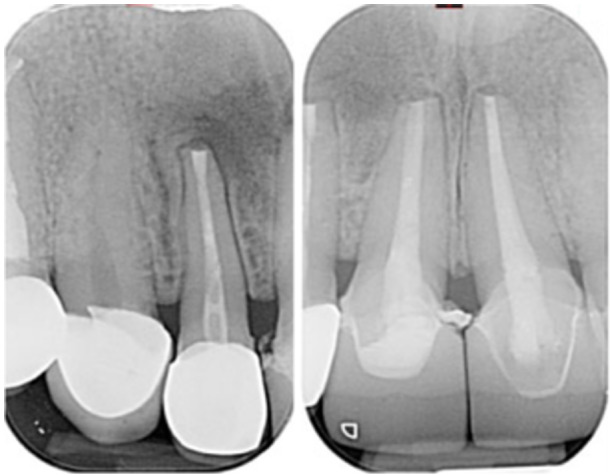

Case 3: The preoperative CBCT and periapical radiograph show an extensive periapical radiolucency involving teeth 11 and 12, with a loss of the labial cortical bone. Apical surgery was initially performed on teeth 11 and 12, but a few months later, tooth 21 required RCT and apical surgery (case 7). At 6 months, marked healing can be seen, evidenced by a reduction in the periapical radiolucency size associated with teeth 11 and 12. Note that the crowns on teeth 11 and 21 have also been replaced with new ones. Cases 3 and 4 are from the same patient but with different L‐PRF preparations.

**Table 9:** Case 4 radiographs in sequence: Tooth 21.

Pre‐op CBCT	Pre‐op IOPA	Post‐op IOPA	Post‐op IOPA (6 months following surgery)
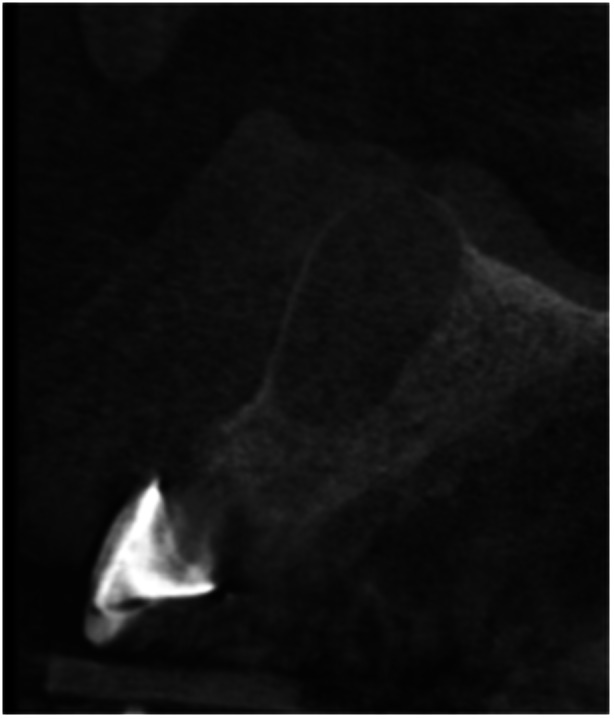	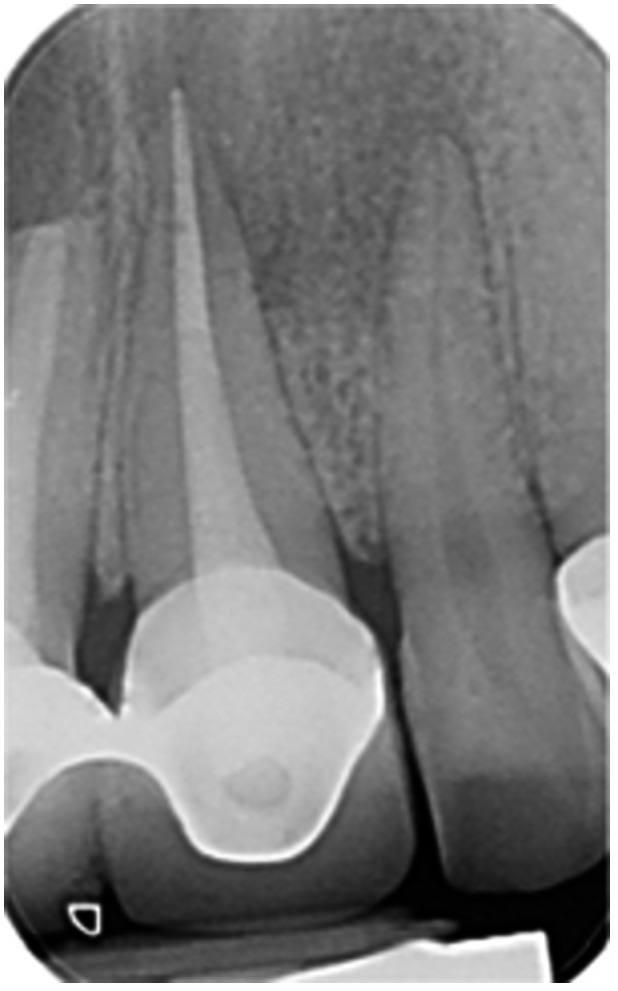	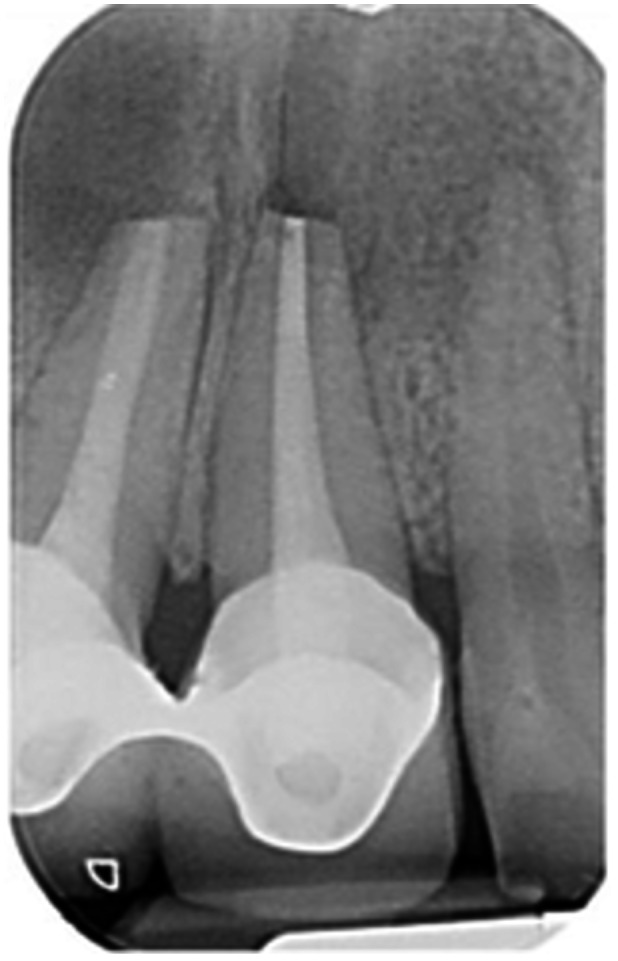	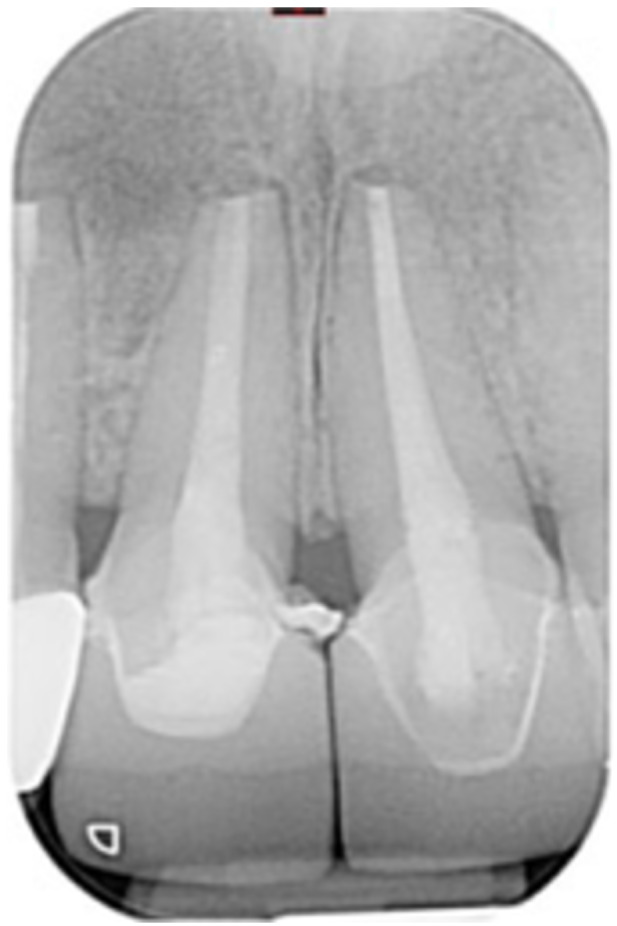

Case 4: The preoperative CBCT and periapical radiograph show an extensive periapical radiolucency associated with tooth 21. Apical surgery was performed on tooth 21 after the completion of a satisfactory nonsurgical root canal treatment on the tooth. At 6 months, marked healing can be seen, evidenced by a reduction in the periapical radiolucency size associated with tooth 21, with almost complete healing noted. Note that the crown on tooth 21 has also been replaced with a new one. Cases 3 and 4 are from the same patient but with different L‐PRF preparations.

**Table 10:** Case 5 radiographs in sequence: Tooth 21.

Pre‐op CBCT	Pre‐op IOPA	Post‐op IOPA	Post‐op IOPA (6 months following surgery)
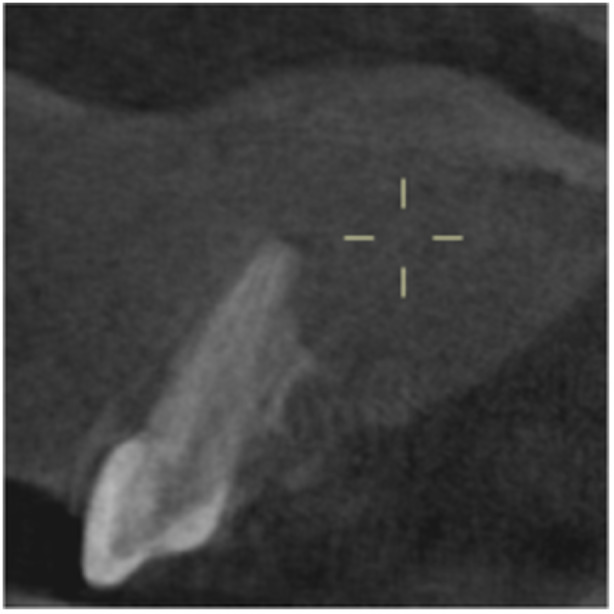	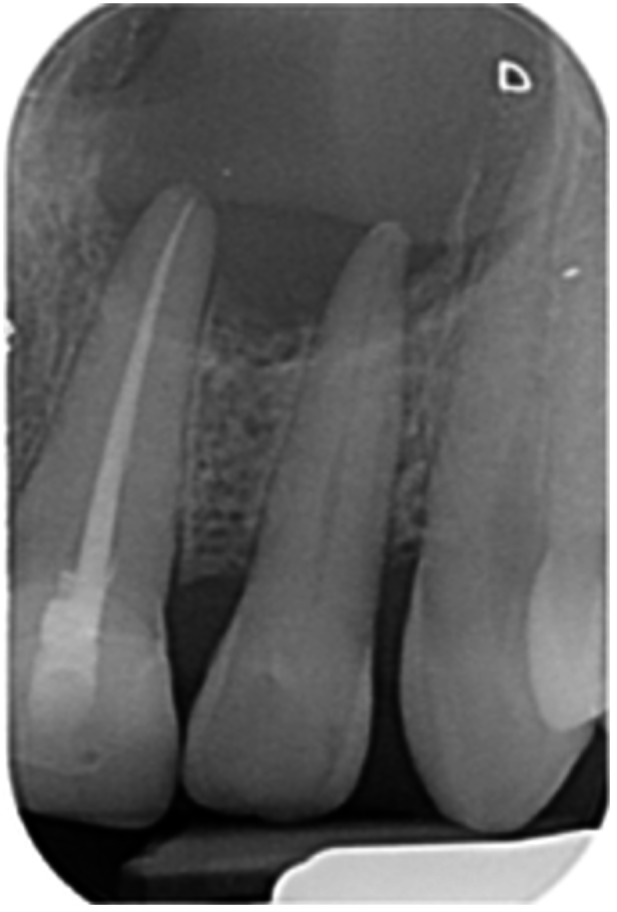	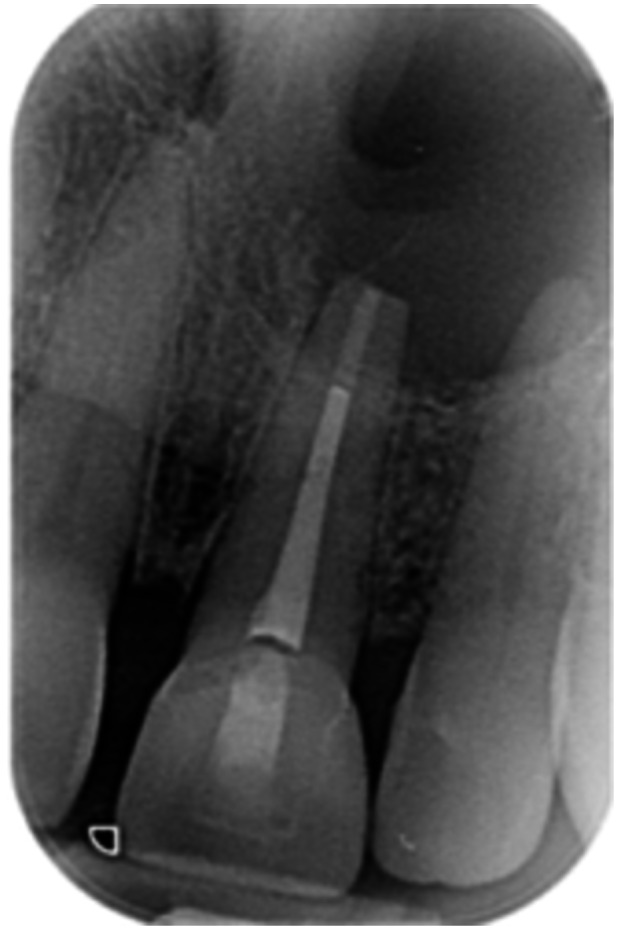	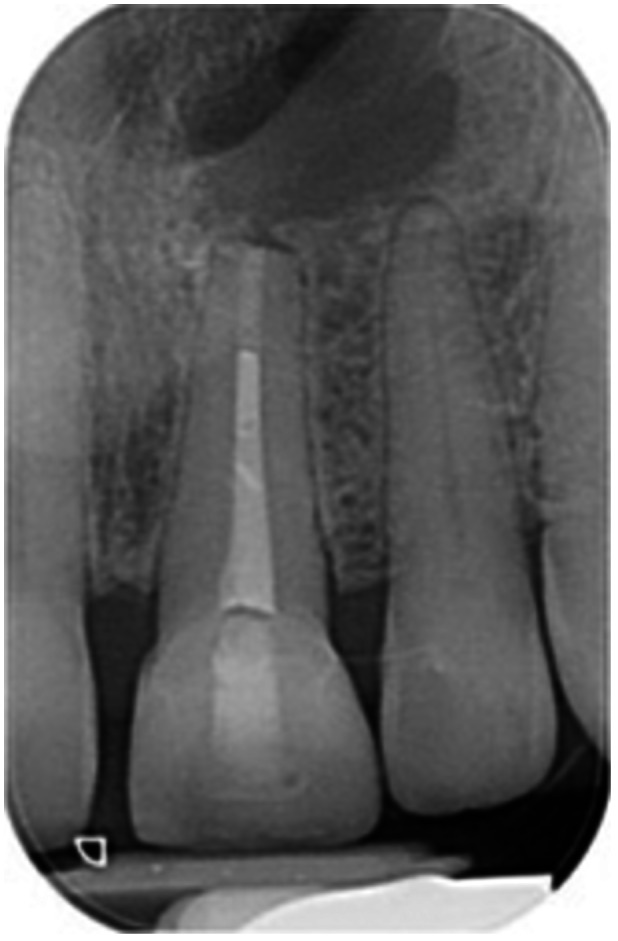

Case 5: The preoperative CBCT and periapical radiograph show an extensive periapical radiolucency associated with tooth 21, which has extended to teeth 22 and 23. Apical surgery was performed on tooth 21 after the completion of a satisfactory nonsurgical root canal retreatment on the tooth. At 6 months, marked healing can be seen, evidenced by a reduction in the periapical radiolucency size associated with tooth 21, with PDL reattachment noted around the roots of teeth 21 and 22.

**Table 11:** Case 6 radiographs in sequence: Teeth 11, 12.

Pre‐op CBCT	Pre‐op IOPA	Post‐op IOPA	Post‐op IOPA (6 months following surgery)
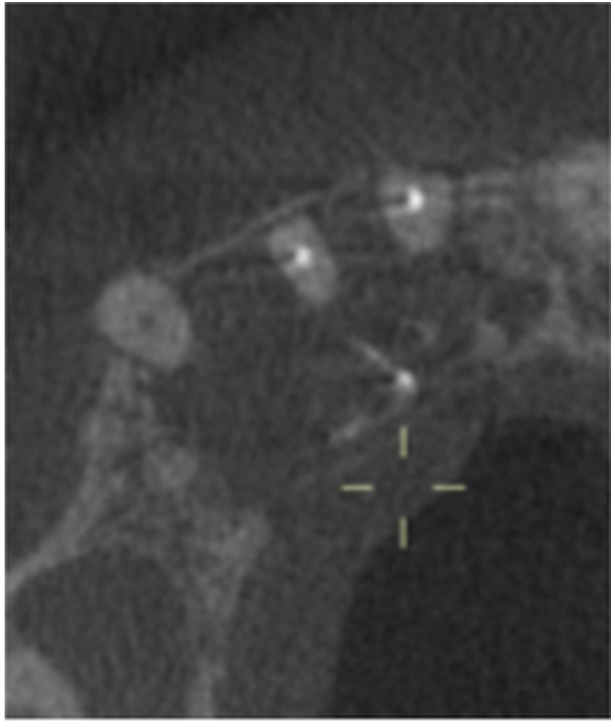 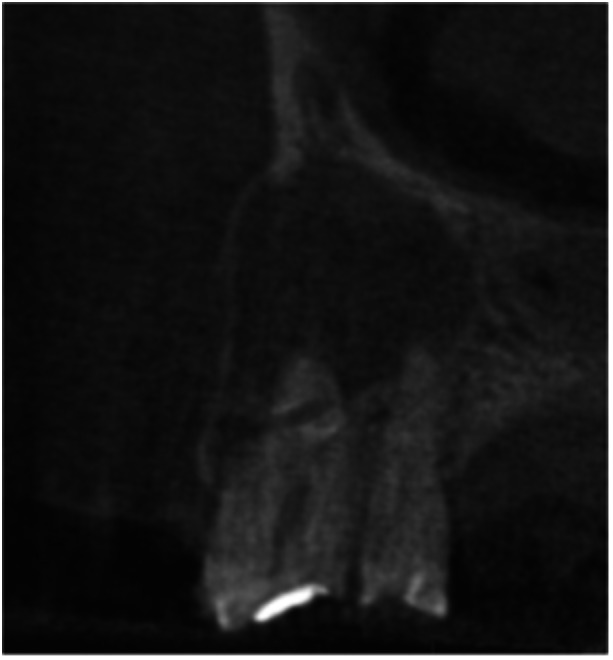	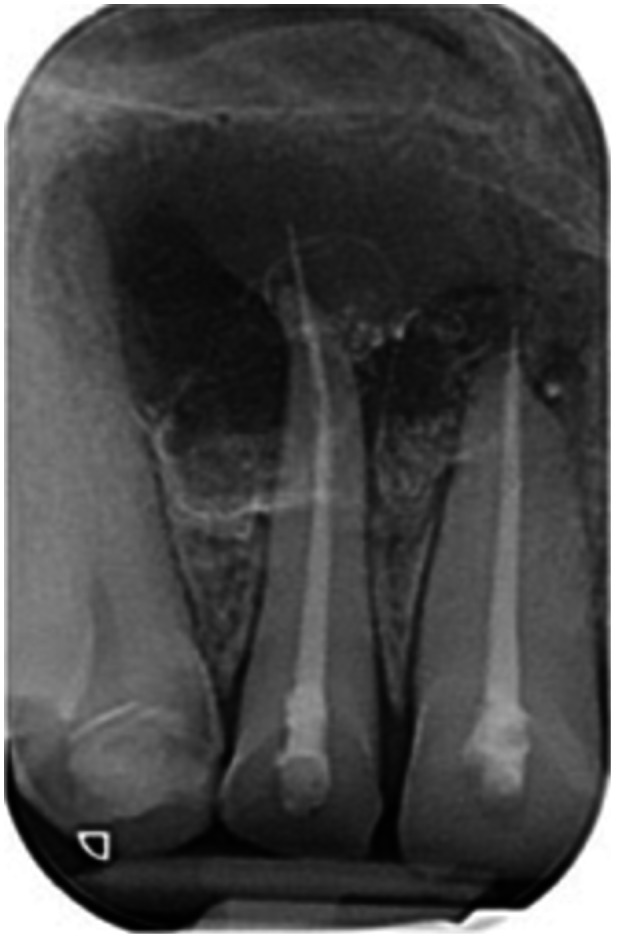	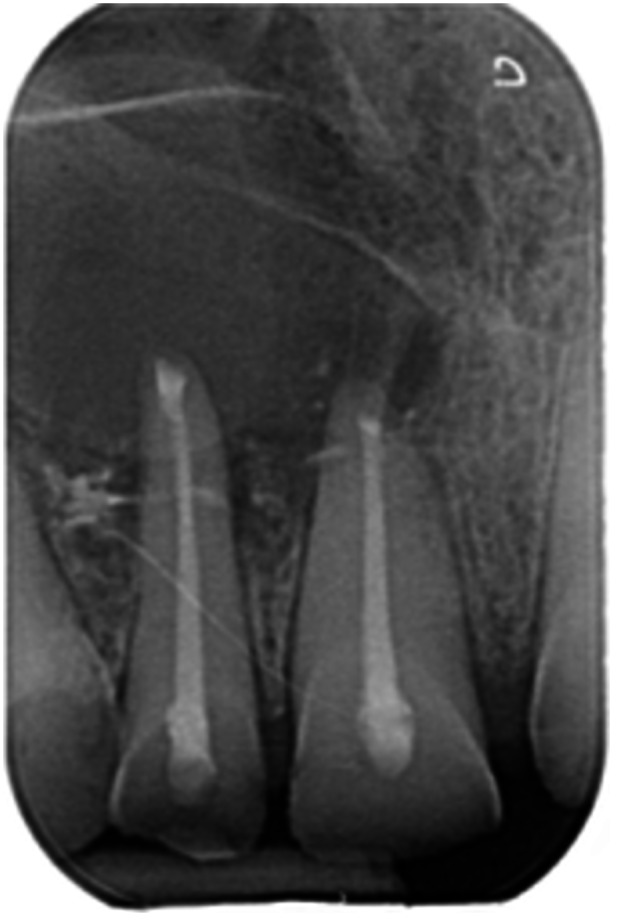	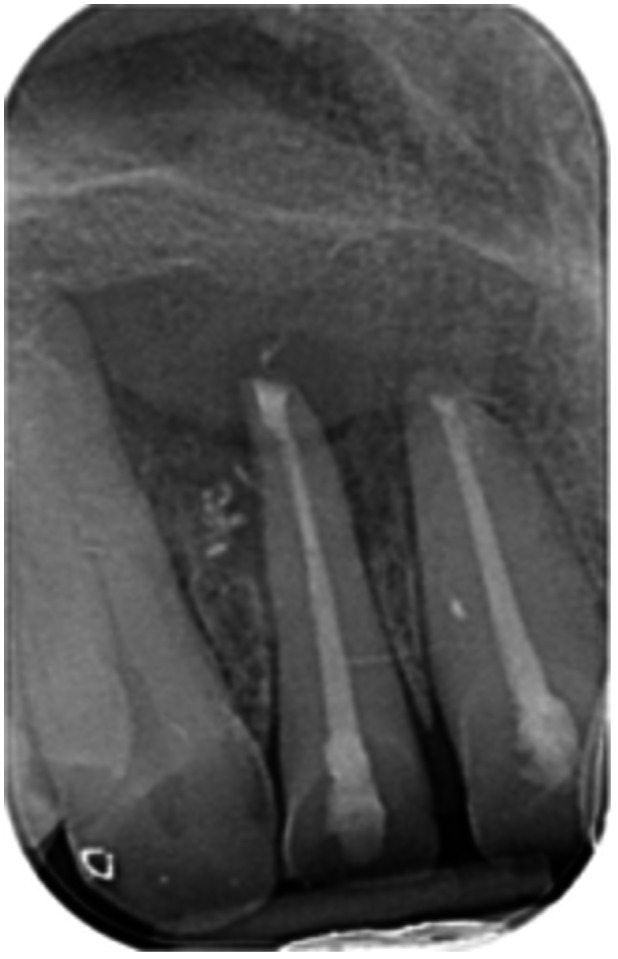

Case 6: The preoperative CBCT and periapical radiograph show an extensive periapical radiolucency associated with teeth 11 and 12, which has extended to tooth 13 as well. Apical surgery was performed on teeth 11 and 12 only, as tooth 13 was deemed vital. At 6 months, marked healing can be seen, evidenced by a reduction in the periapical radiolucency size associated with teeth 11 and 12, with PDL reattachment noted around the root of tooth 11.

**Table 12:** Case 7 radiographs in sequence: Tooth 12.

Pre‐op CBCT	Pre‐op IOPA	Post‐op IOPA	Post‐op IOPA (6 months following surgery)
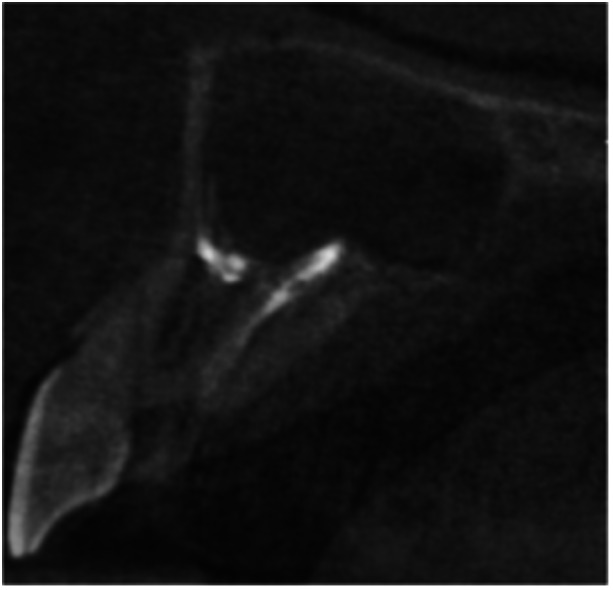 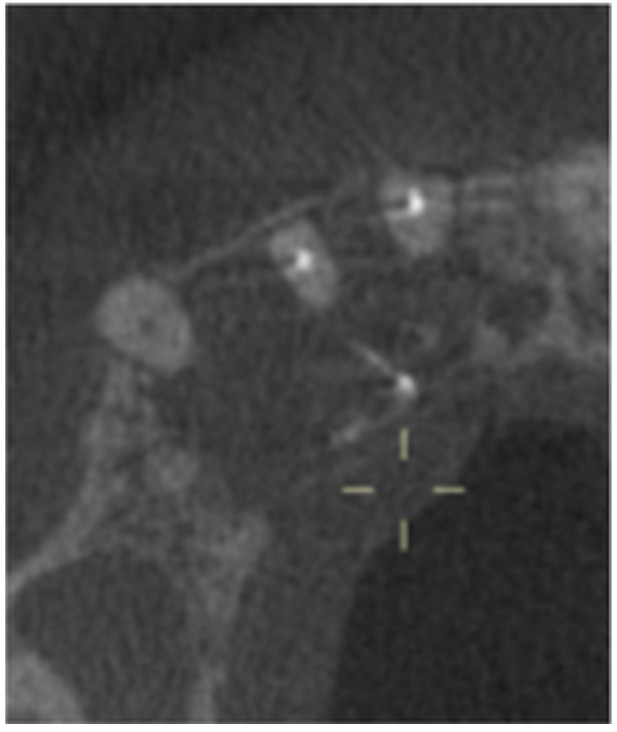	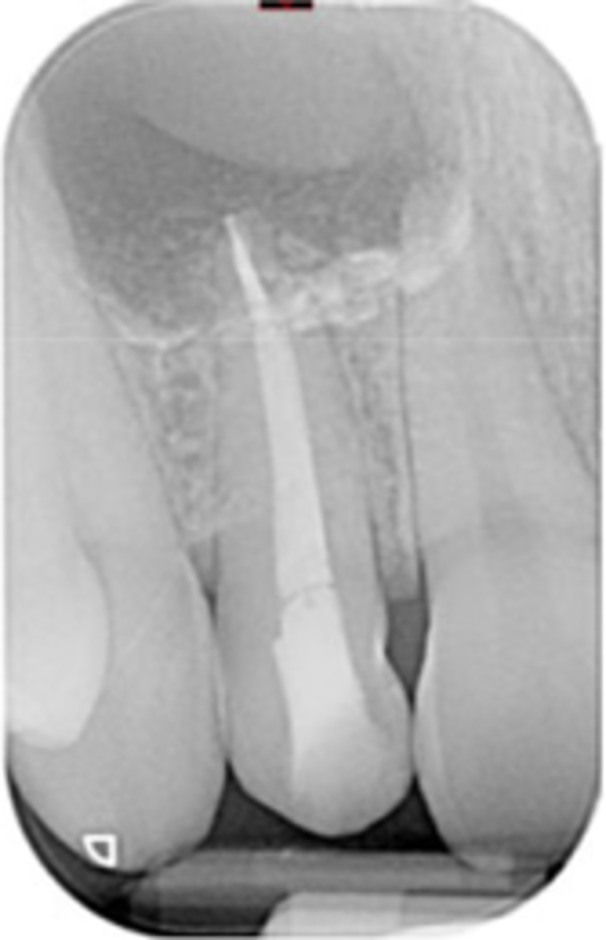	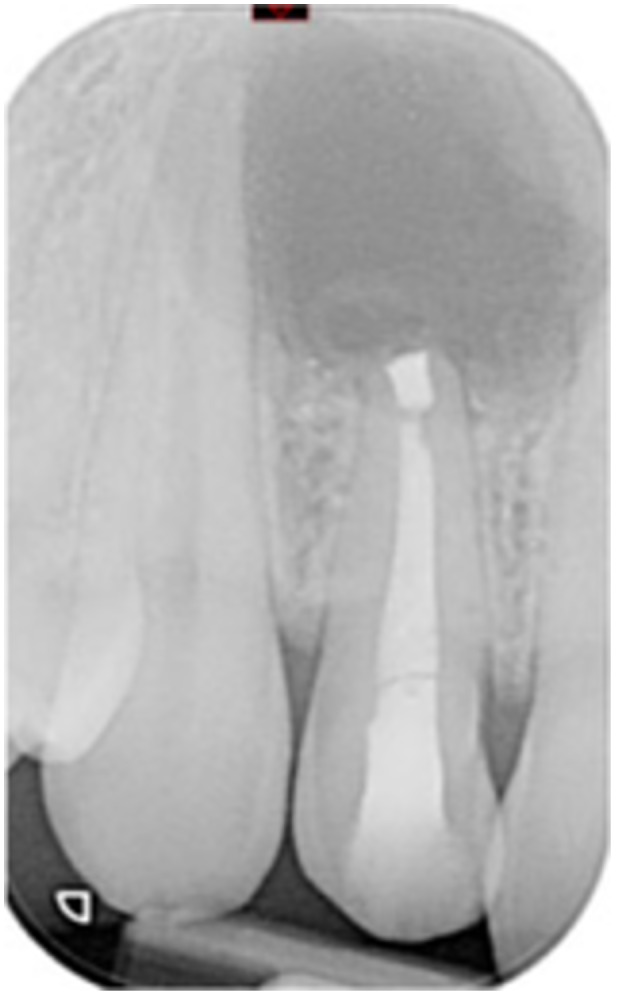	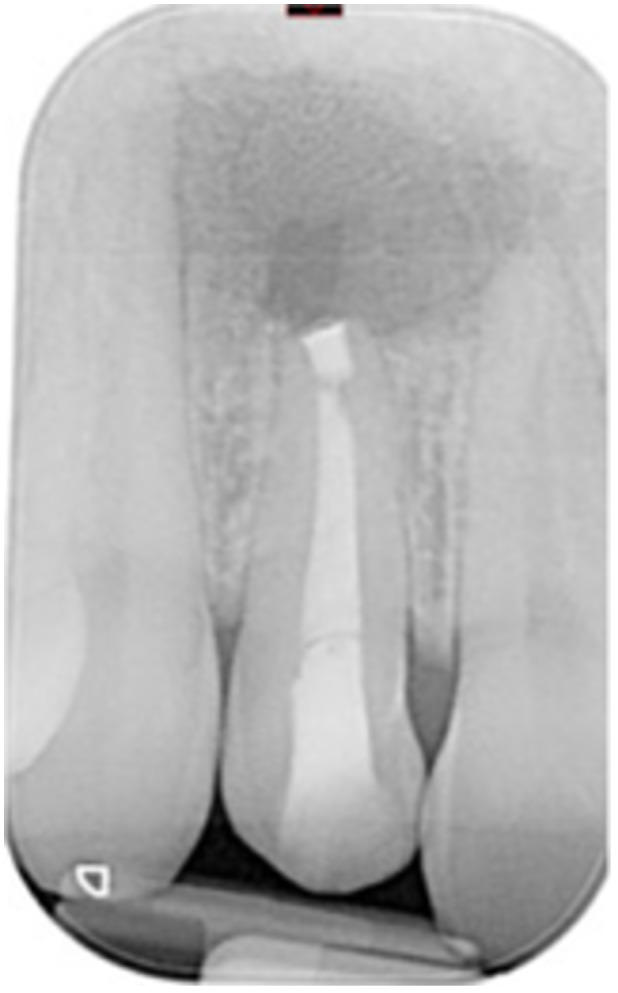

Case 7: The preoperative CBCT and periapical radiograph show an extensive periapical radiolucency associated with tooth 12, which has extended to teeth 11 and 13. Apical surgery was performed on tooth 12 only, as teeth 11 and 13 were deemed vital. At 6 months, marked healing can be seen, evidenced by a reduction in the periapical radiolucency size associated with tooth 12.

**Table 13:** Case 8 radiographs in sequence: Tooth 13.

Pre‐op CBCT	Pre‐op IOPA	Post‐op IOPA	Post‐op IOPA (6 months following surgery)
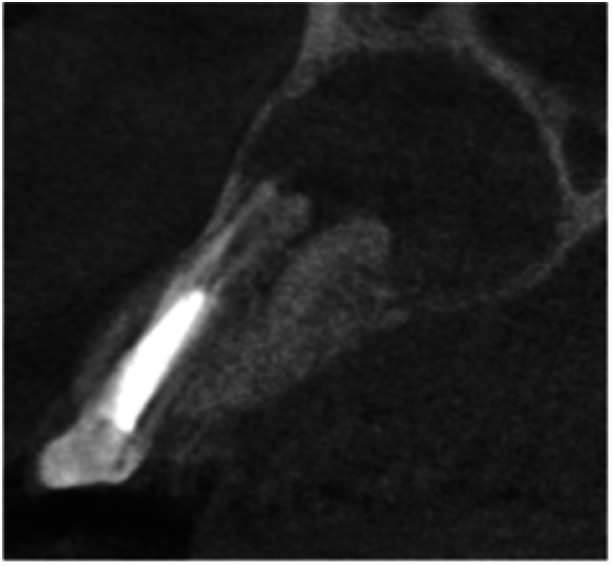 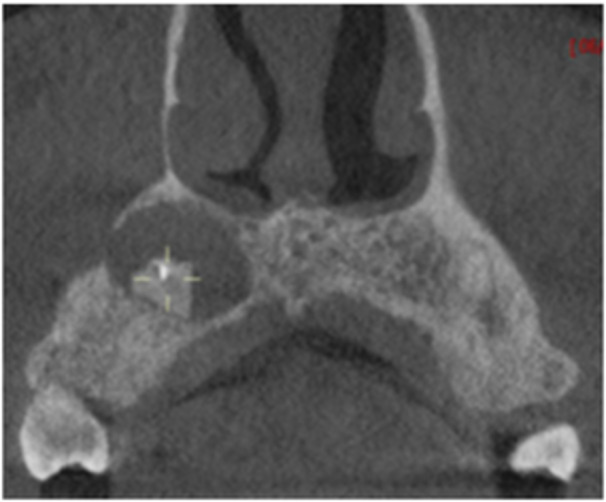 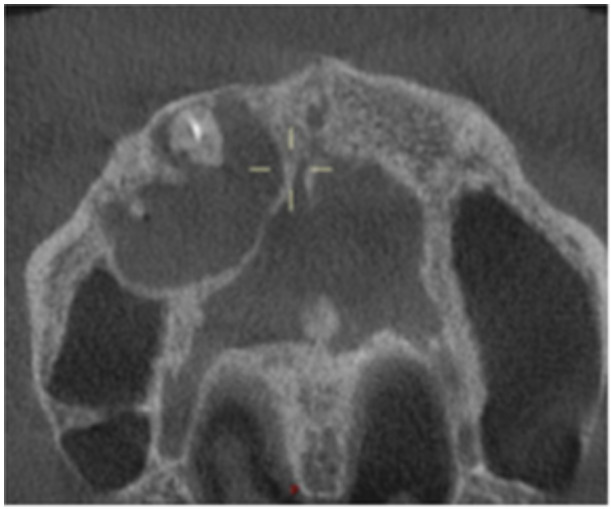	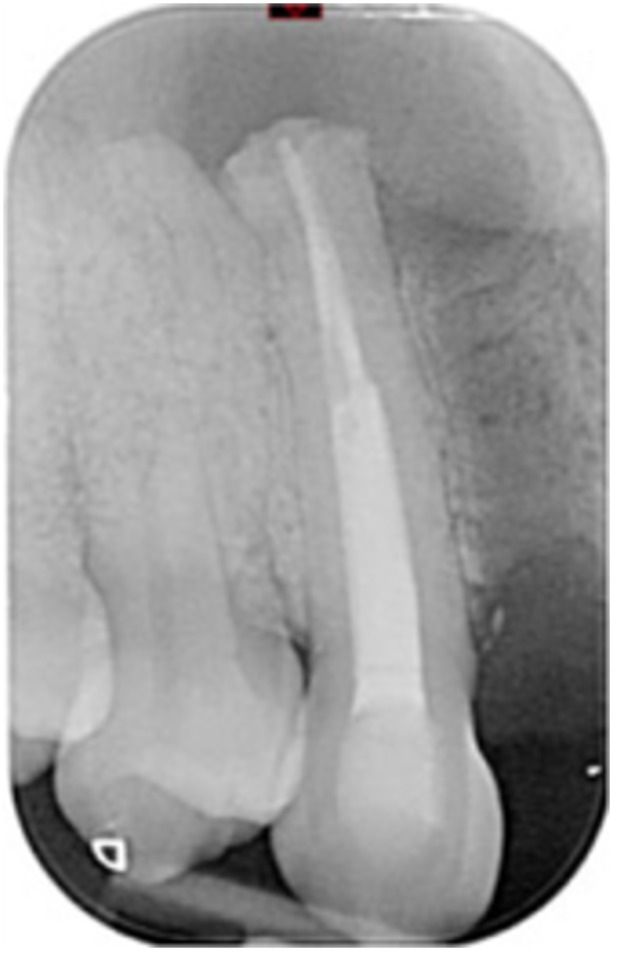	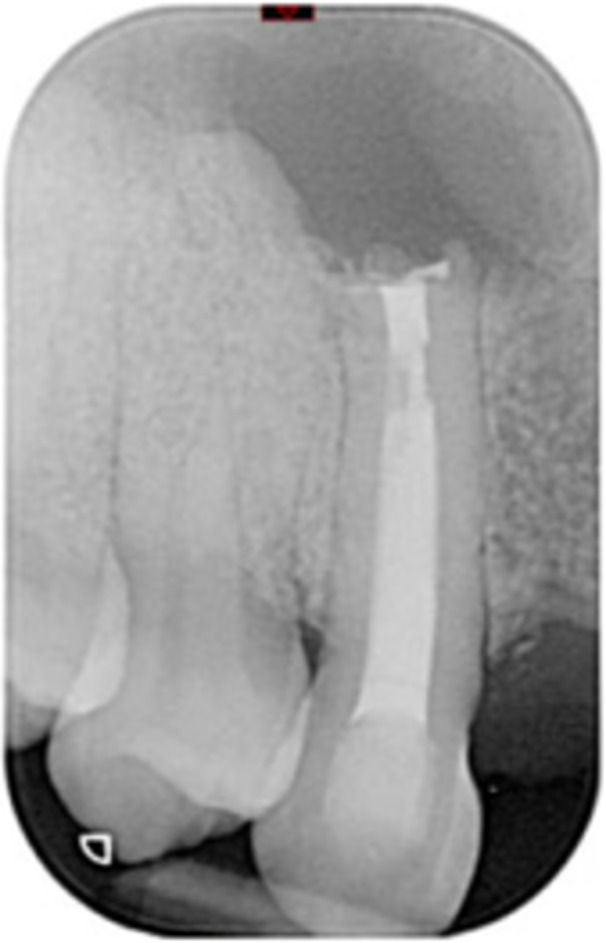	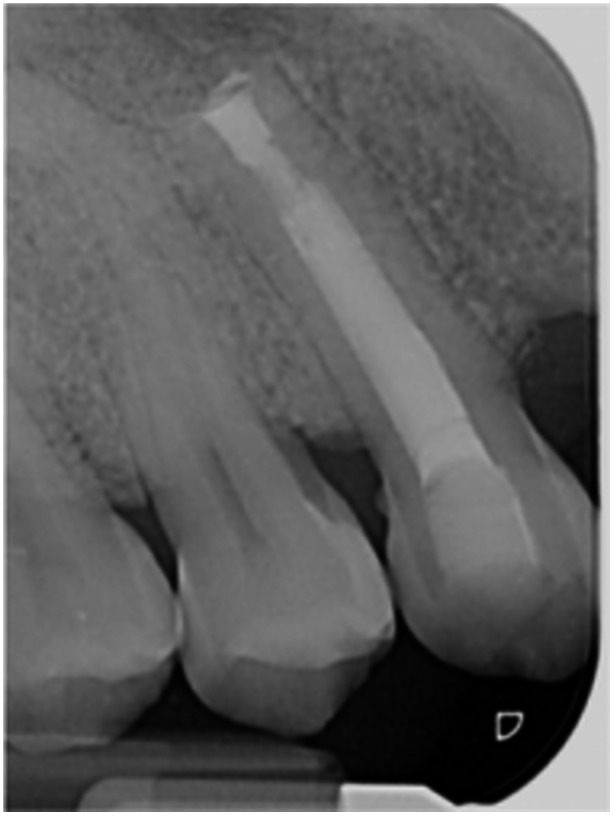

Case 8: The preoperative CBCT and periapical radiograph show an extensive periapical radiolucency associated with tooth 13, which has thinned the labial and palatal cortical plates. Apical surgery was performed on tooth 13. At 6 months, complete healing is evident.

**Table 14:** Case 9 radiographs in sequence: Tooth 12.

Pre‐op CBCT	Pre‐op IOPA	Post‐op IOPA	Post‐op IOPA (6 months following surgery)
No CBCT taken	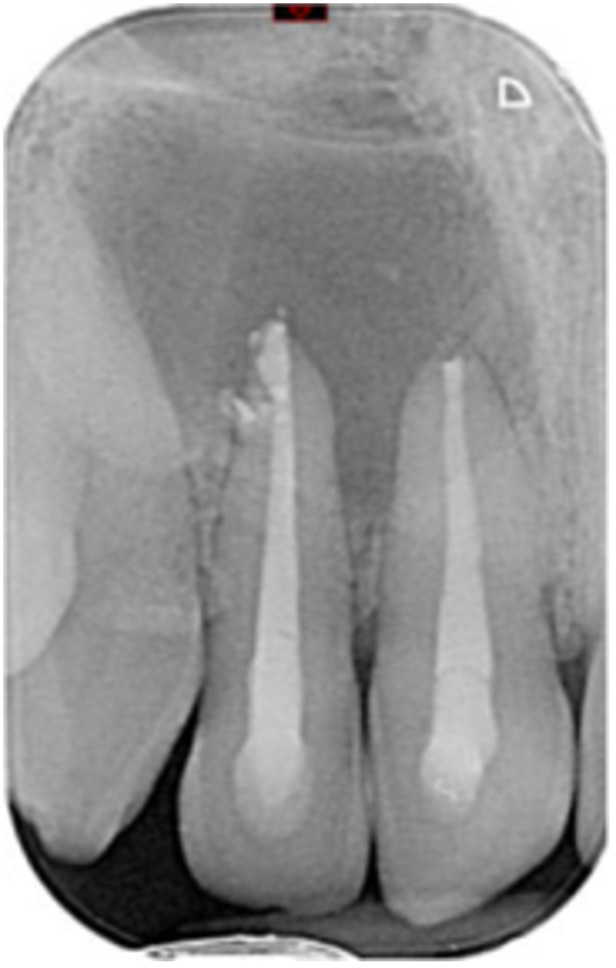	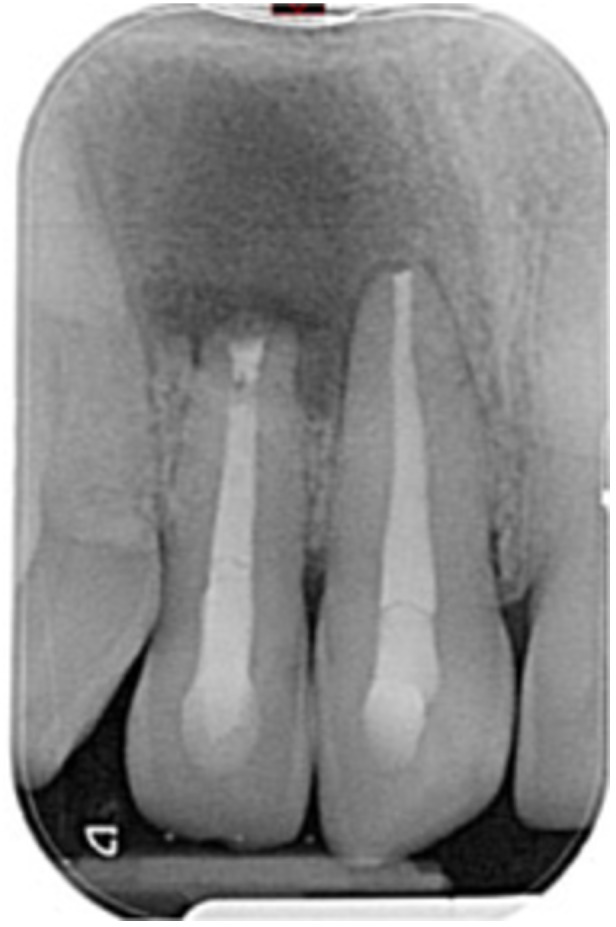	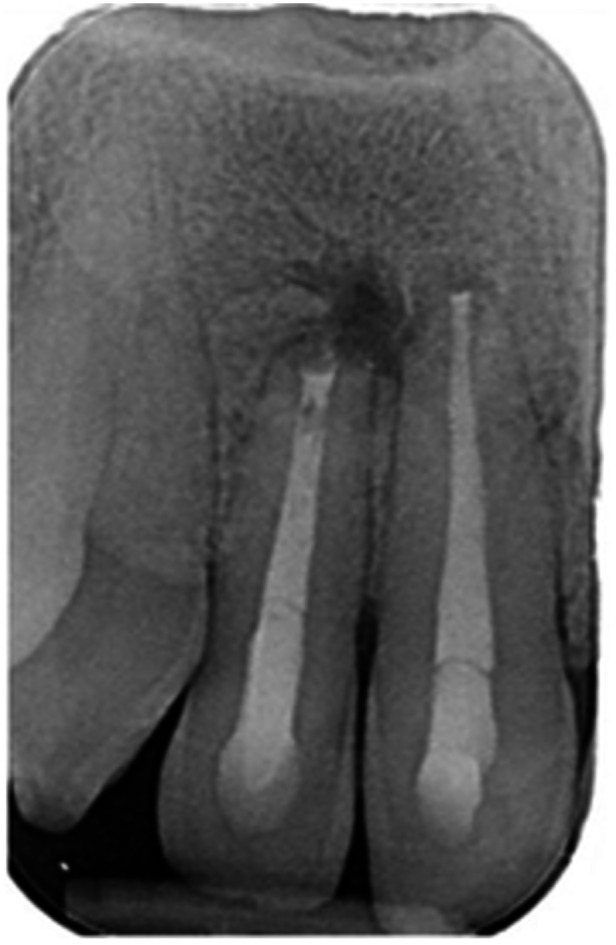

Case 9: The preoperative periapical radiograph reveals a large periapical radiolucency associated with tooth 12, extending to tooth 11. Apical surgery was performed on tooth 12 only. At the 6‐month follow‐up, complete PDL reattachment is observed on tooth 11, while tooth 12 shows incomplete healing. However, there is a significant reduction in the size of the periapical radiolucency.

**Table 15:** Case 10 radiographs in sequence: Tooth 22.

Pre‐op CBCT	Pre‐op IOPA	Post‐op IOPA	Post‐op IOPA (6 months following surgery)
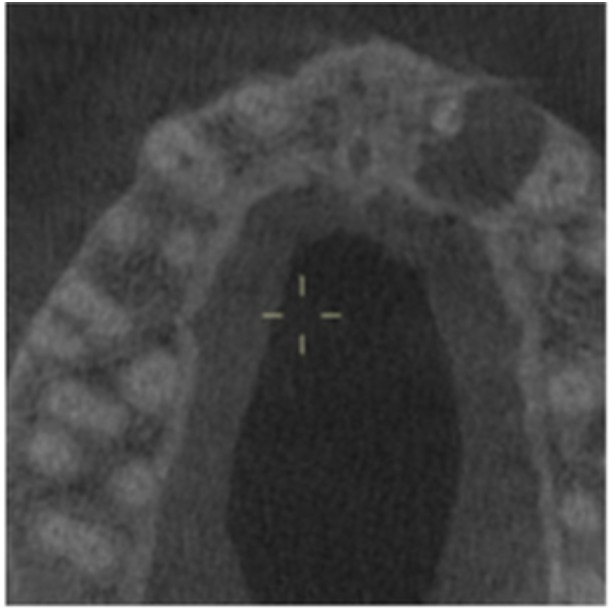 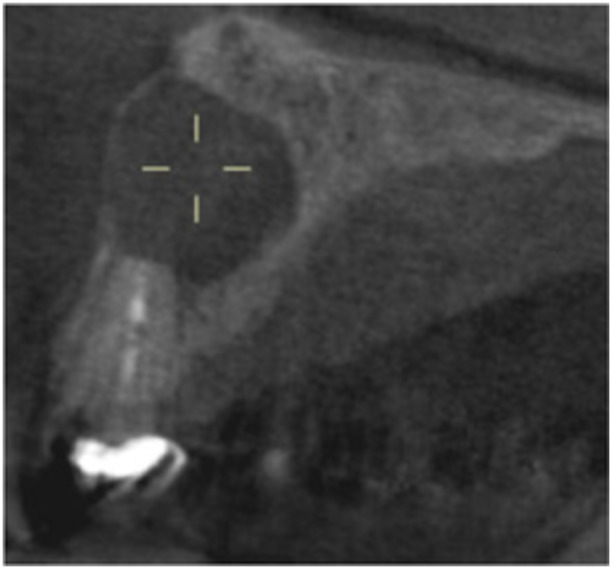	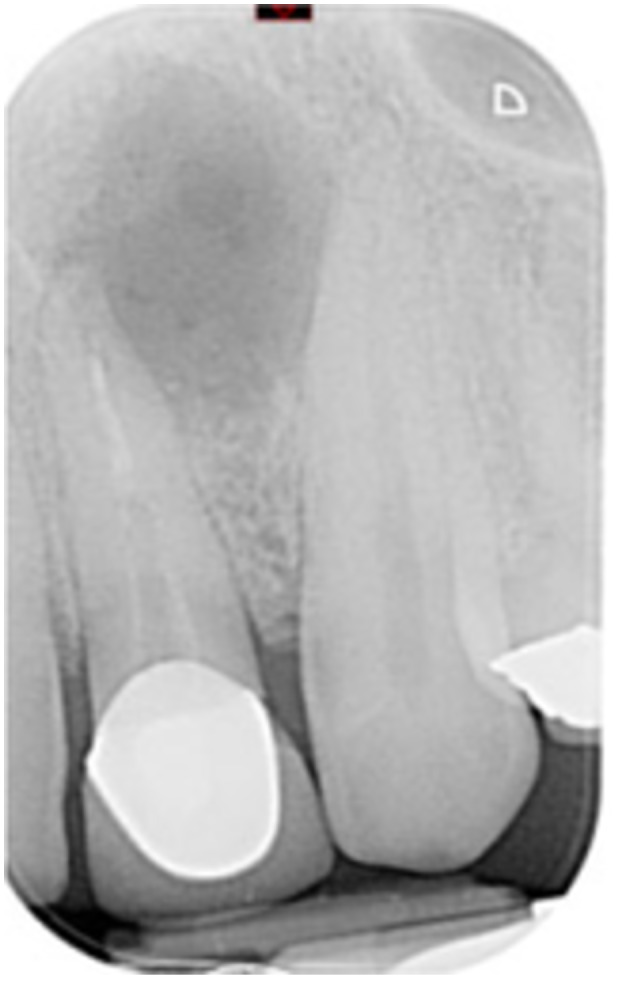	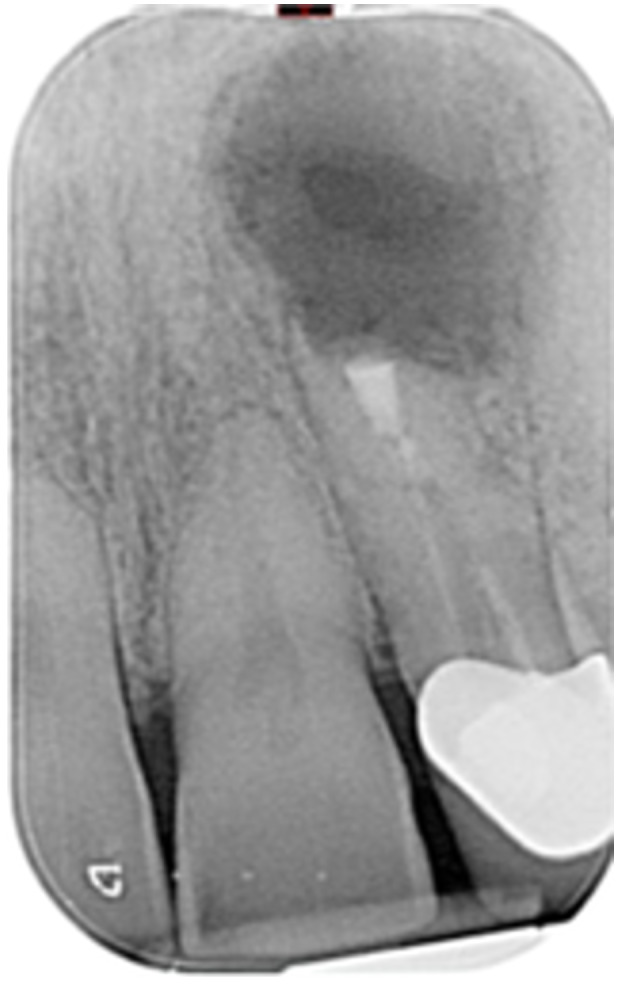	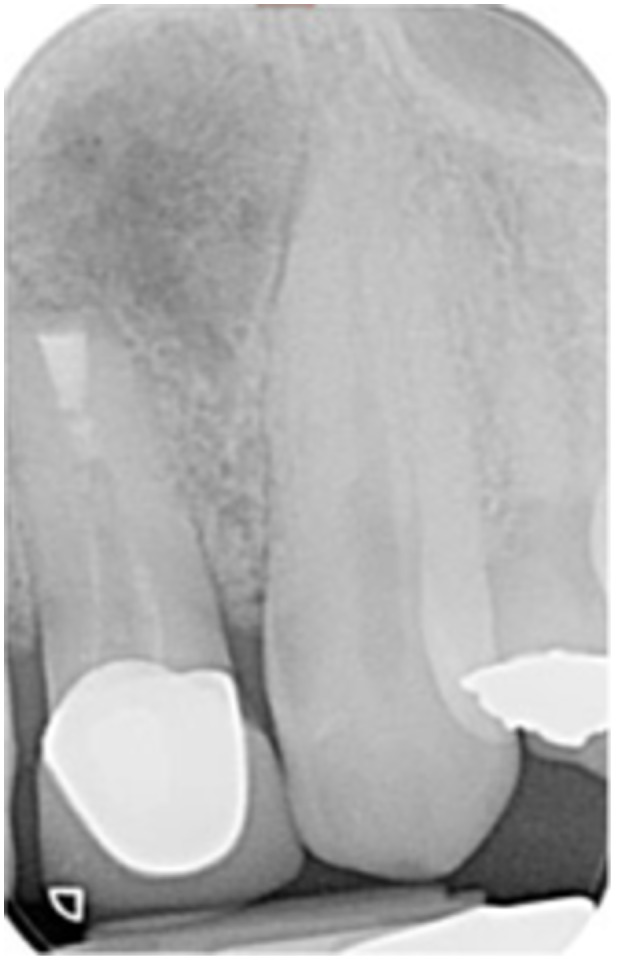

Case 10: The preoperative CBCT and periapical radiograph reveal a large periapical radiolucency associated with tooth 22, which has thinned the labial cortical plate. Apical surgery was performed on tooth 22 without prior nonsurgical root canal retreatment due to the presence of a fiber post, complicating the nonsurgical root canal retreatment. Nonetheless, the crown had satisfactory margins. At the 6‐month follow‐up, a significant reduction in the size of the periapical radiolucency can be seen.

**Table 16:** Case 11 radiographs in sequence: Tooth 11.

Pre‐op CBCT	Pre‐op IOPA	Post‐op IOPA	Post‐op IOPA (6 months following surgery)
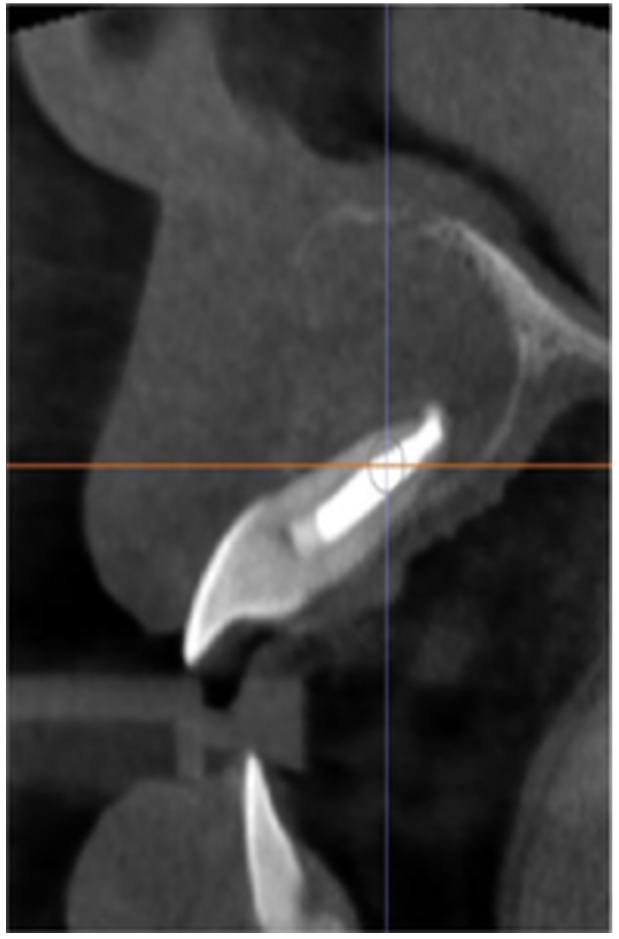	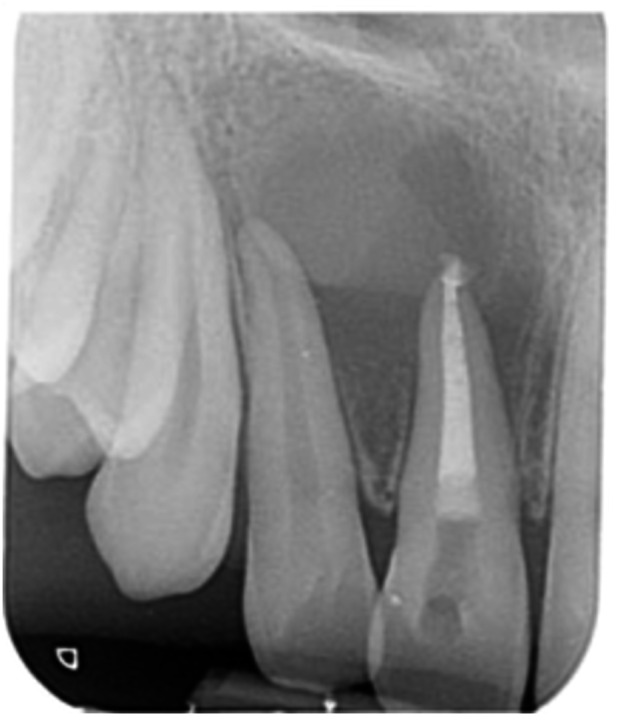	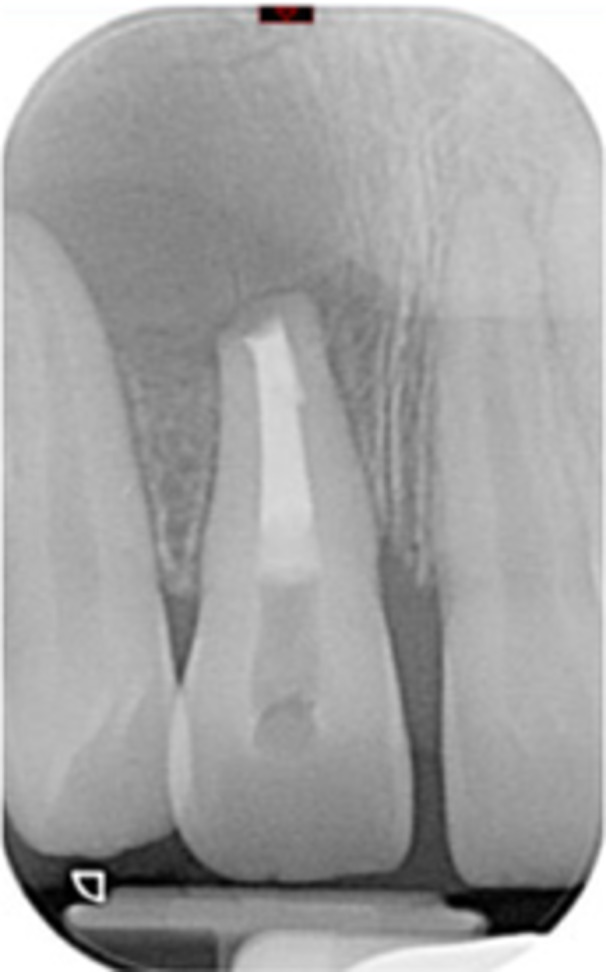	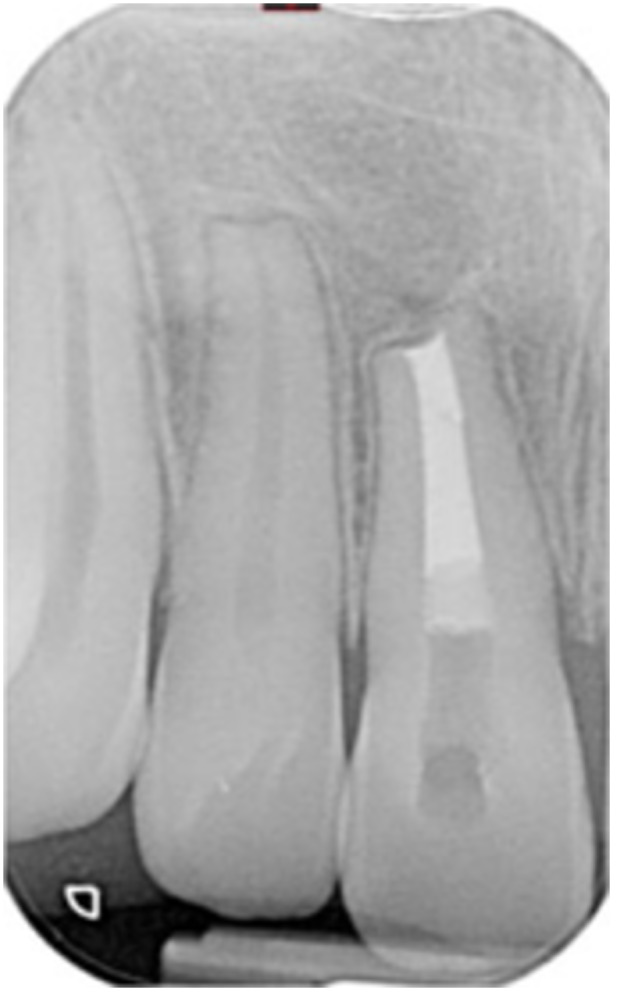

Case 11: The preoperative CBCT and periapical radiograph show an extensive periapical radiolucency associated with tooth 11, which has thinned the labial cortical plate and has caused displacement of tooth 12. Apical surgery was performed on tooth 11 following satisfactory nonsurgical root canal treatment. At the 6‐month follow‐up, complete healing is observed, and tooth 12 has returned to its upright position.

**Table 17:** Case 12 radiographs in sequence: Teeth 32, 31, 41, and 42.

Pre‐op CBCT	Pre‐op IOPA	Post‐op IOPA	Post‐op IOPA (6 months following surgery)
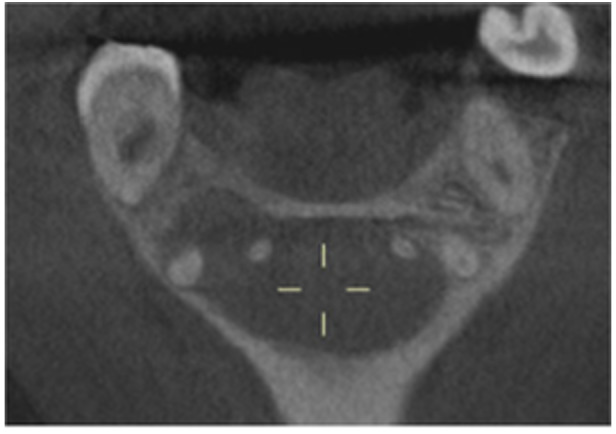	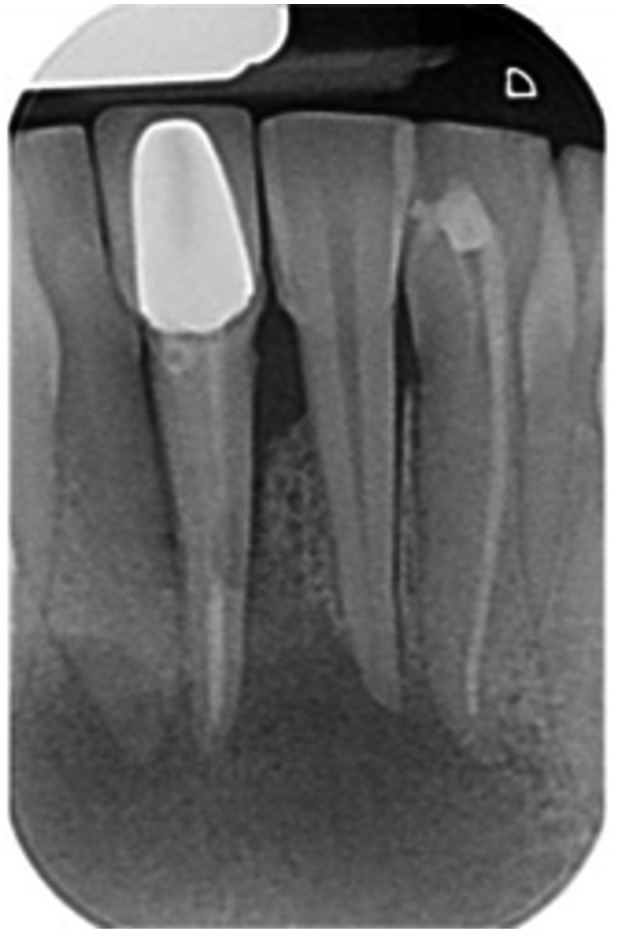	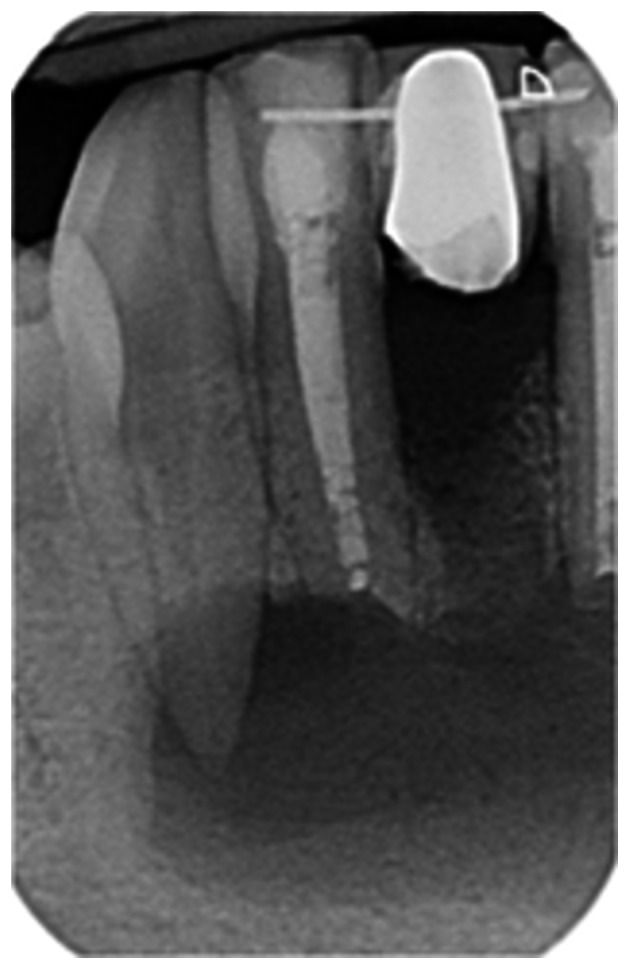	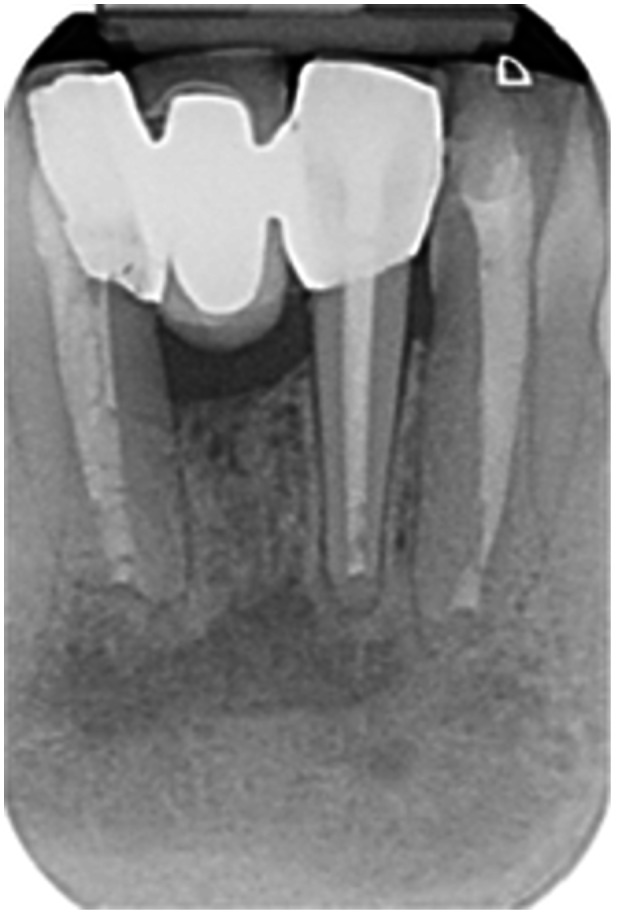

Case 12: The preoperative CBCT and periapical radiograph show an extensive periapical radiolucency associated with teeth 32, 31, 41, and 42. Tooth 41 was eventually extracted due to vertical root fracture, nonsurgical root canal treatment performed on teeth 42 and 31, and nonsurgical root canal retreatment on tooth 32. Due to persistent infection, apical surgery was performed on teeth 42, 31, and 32. At the 6‐month follow‐up, a significant reduction in the size of the periapical radiolucency can be seen, with PDL reattachment noted at teeth 42, 31, and 32 and complete healing of the apical tissues of tooth 32.

**Table 18:** Case 13 radiographs in sequence: Tooth 11.

Pre‐op CBCT	Pre‐op IOPA	Post‐op IOPA	Post‐op IOPA (4 months following surgery)
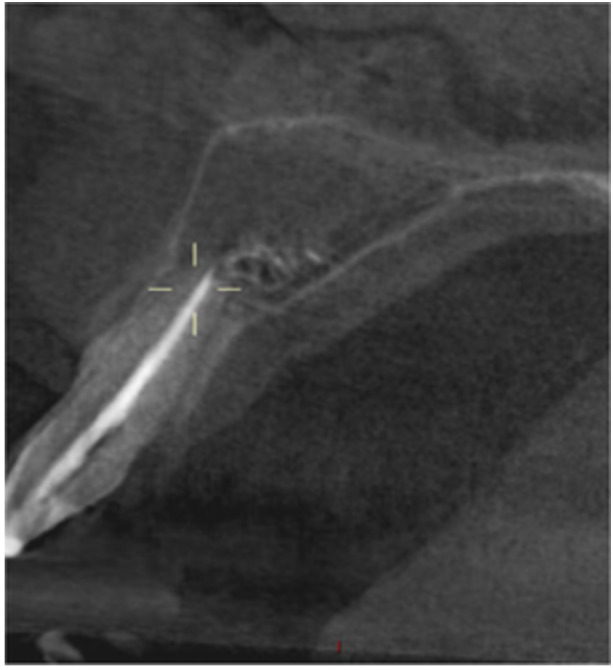 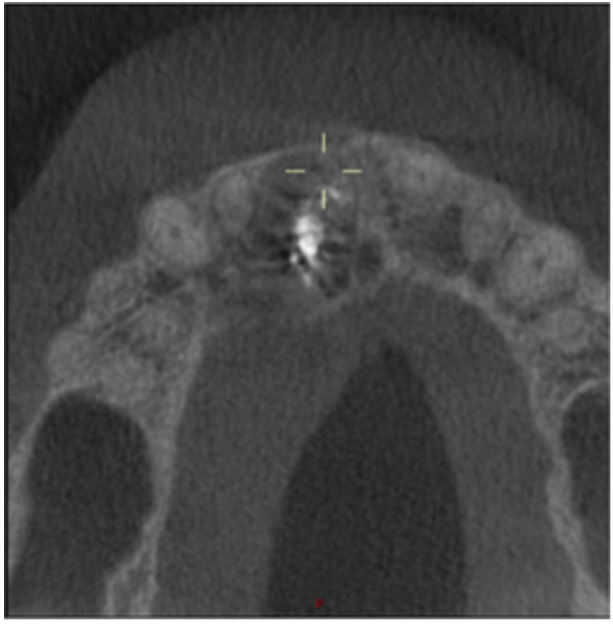 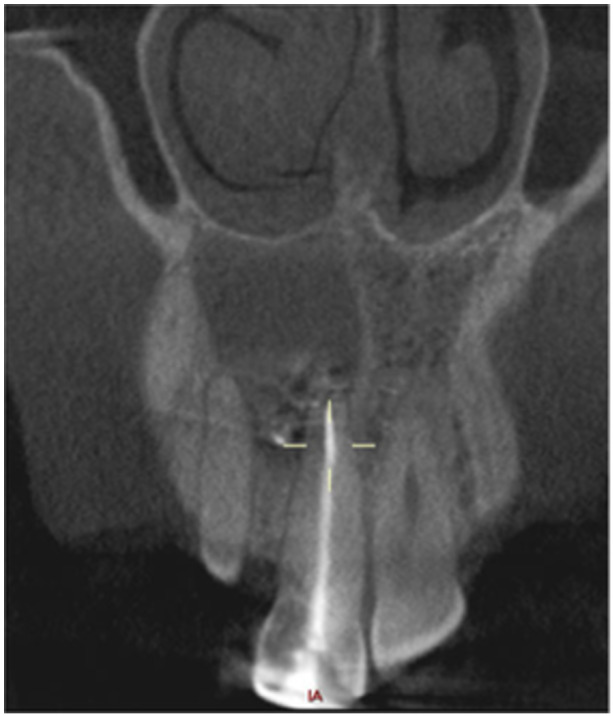	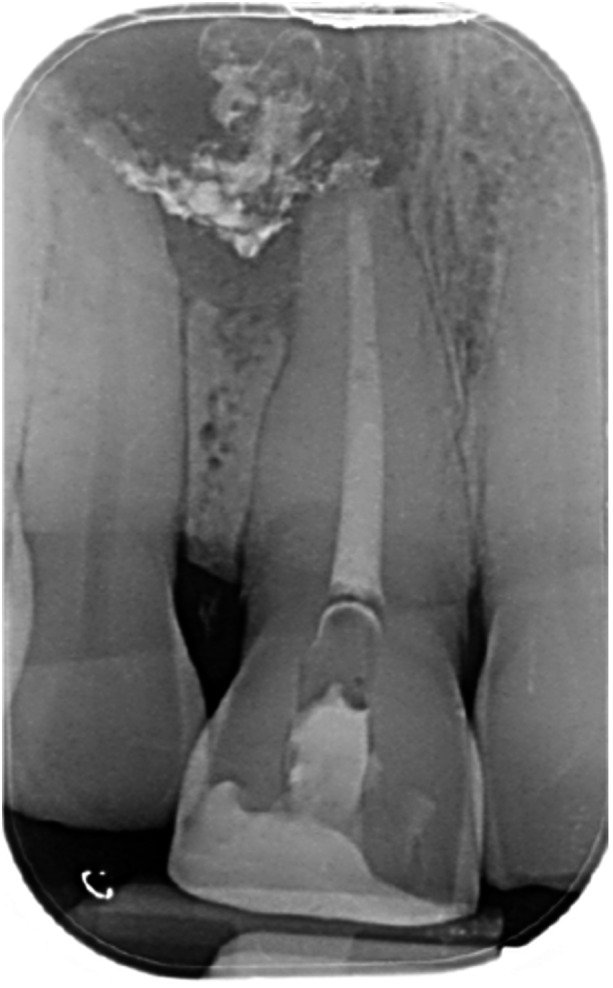	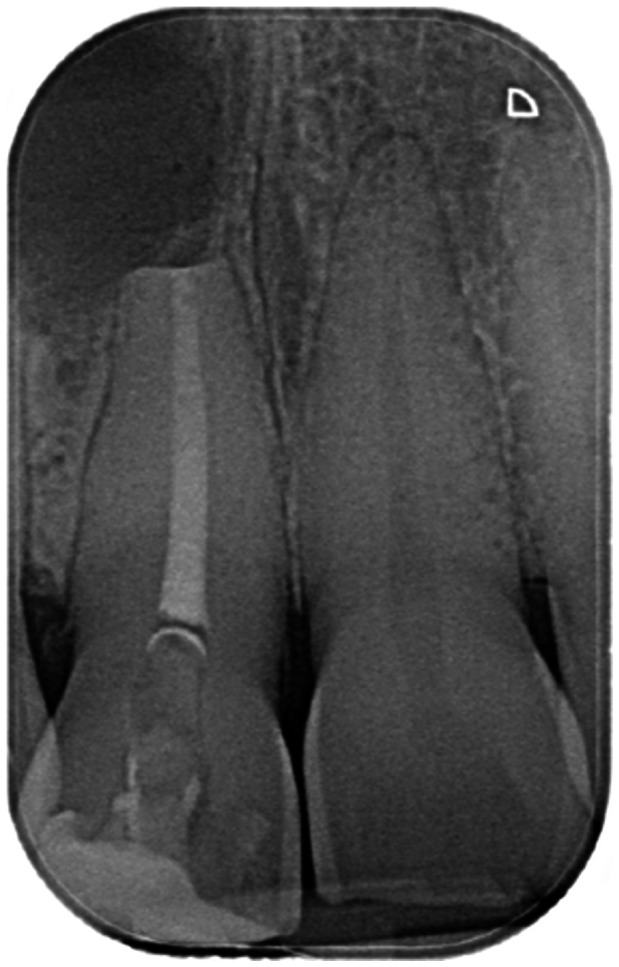	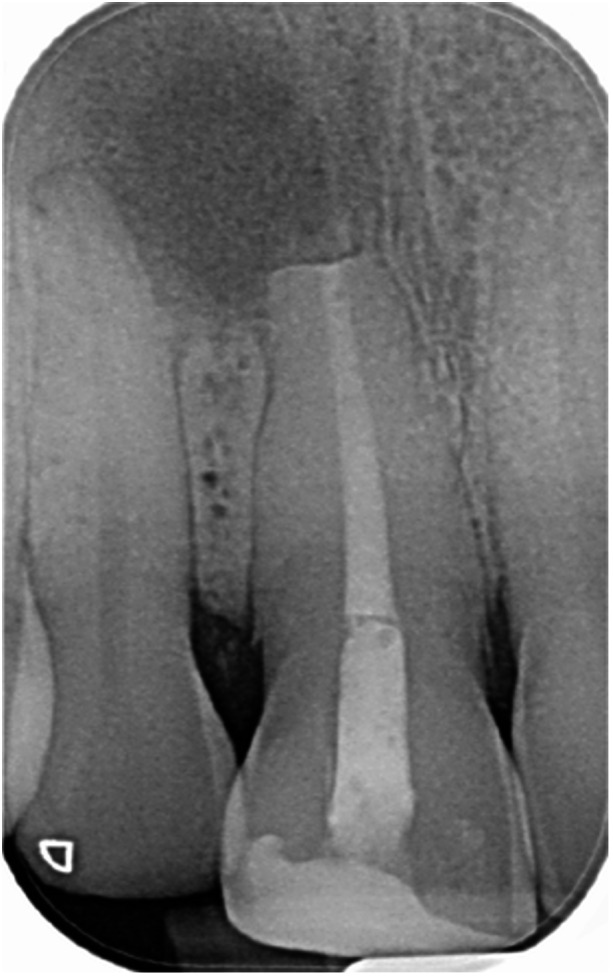

Case 13* (review was done at 4 months): The preoperative CBCT and periapical radiograph show an extensive periapical radiolucency associated with tooth 11, which has thinned the labial and palatal cortical plates. Apical surgery was performed on tooth 11 after the completion of a satisfactory nonsurgical root canal retreatment on the tooth. At 4 months, marked healing can be seen, evidenced by a reduction in the periapical radiolucency size associated with tooth 11.

Among the 13 cases analyzed, none of the patients experienced adverse effects from the application of L‐PRF at the surgical site, whether in the form of membranes or L‐PRF plugs. All cases demonstrated clinical benefits, evidenced by a reduction in apical radiolucency size and the absence of pain, except for mild discomfort reported by two patients at 24 h post‐surgery.

## 4 Discussion

Platelet concentrates have evolved over two generations. The first generation, introduced in the 1990s, included PRP and platelet‐rich growth factor (PGRF), requiring additives like bovine thrombin or calcium chloride (CaCl_2_). These were time‐consuming to prepare and lacked leukocytes. The second‐generation L‐PRF, developed by Choukroun in 2001, is a simplified, additive‐free preparation that gradually releases growth factors over 7–14 days, enhancing regeneration (Dohan Ehrenfest et al. 2014). While widely used in periodontal and implant surgery, its application in apical surgery remains limited. In endodontic microsurgery, regeneration is preferable to repair, and Tsesis et al. (2011) recommended guided tissue regeneration for lesions > 10 mm or through‐and‐through defects. L‐PRF significantly improves soft and hard tissue healing (Pinto et al. 2017). Singh et al. (2013) reported complete apical healing with bone regeneration at 6 months post‐surgery. All cases showed a significant reduction in the size of the periapical radiolucency at the 6‐month review, evidenced by the hard tissue deposition as seen on the periapical radiographs. Based on the findings observed in this case series, there was no correlation between the rate of healing and the age, gender, preoperative size of the periapical radiolucency, or histopathological diagnosis, but this was done based on observation and not through a statistical analysis. However, the two cases that showed complete healing of the apical tissues involved patients without any medical conditions, nonsmokers, and both were in the anterior maxillary region. Whether the number of L‐PRF membranes and/or L‐PRF plugs used had an impact on the outcome at 6 months could not be evaluated, as some clinicians did not record this information.

Based on the existing L‐PRF preparation protocol, which involves centrifugation of blood for 12 min at 2700 rpm, stable L‐PRF membranes were produced, aiding in periapical wound healing. This approach is particularly beneficial for cases with large periapical lesions (> 10 mm). The incorporation of L‐PRF in endodontic microsurgery aims to accelerate wound healing and enhance bone formation rather than scar tissue formation, especially when the apical radiolucency exceeds 5 mm. According to the Toronto Study (Wang et al. 2004), the success rate of apical surgery decreases as the size of the apical radiolucency increases (> 5 mm). This decline may be due to healing with scar tissue, which appears radiolucent on radiographs and may be misinterpreted as uncertain healing rather than true apical healing, thereby reducing reported success rates. During wound healing, the cells with the fastest migration rate dominate the process (Meschi et al. 2018). Fibroblasts, which arrive earlier, initiate the deposition of type III collagen, followed by type I collagen as early as day 2, whereas osteoblasts are not present until later (Harrison and Jurosky 1991). In large apical lesions, fibroblasts often outcompete osteoblasts, leading to scar tissue formation. Without L‐PRF, healing relies solely on localized growth factors. L‐PRF exhibits osteogenic, osteoconductive, and osteoinductive properties (Choukroun et al. 2001). Consequently, the use of L‐PRF in apical tissues may facilitate bone regeneration visible on radiographs, which could account for an increased documented success rate. Choukroun et al. (2006) also reported that L‐PRF reduces healing time by half, from 8 months to 4 months. Given these benefits, L‐PRF presents a promising adjunct in endodontic microsurgery. However, further high‐quality studies are needed to establish definitive conclusions. As a proposed protocol, L‐PRF may be particularly valuable in apical surgery cases involving periapical lesions larger than 10 mm, especially in through‐and‐through defects.

Apical surgery was performed in the cases included in this case series due to persistent signs and symptoms of infection and inflammation despite satisfactory root canal treatment or retreatment. The majority of cases were diagnosed with radicular cysts (76.9%), which are known to have a limited response to nonsurgical root canal treatment alone. It has been reported that periapical radiolucency larger than 5 mm significantly reduces the success rate of nonsurgical treatment (Ng et al. 2008). Specifically, with every millimeter increase in the size of apical radiolucency, the odds of successful nonsurgical treatment decrease by 14% (Ng et al. 2011). Given this, apical surgery becomes a necessary intervention when lesions persist despite optimal root canal treatment (Royal College of Surgeons 2020). Nair highlighted that persistent apical periodontitis is primarily attributed to intraradicular infection, reinforcing the need for thorough disinfection through nonsurgical root canal treatment before considering apical surgery (Nair 2006). However, extra‐radicular infections, cystic lesions (occurring in 6%–55% of cases), and foreign body reactions (31%) are also significant contributors to persistent periapical pathology, none of which respond to nonsurgical therapy alone (Nair 2006). In such instances, surgical intervention is necessary to achieve complete resolution of the lesion, further justifying the treatment approach for the cases included in this case series. In addition to addressing persistent pathology, the application of L‐PRF during apical surgery has been shown to enhance healing by promoting angiogenesis, reducing inflammation, and stimulating tissue regeneration. Given the challenges associated with the healing of large periapical lesions, especially cystic lesions, the adjunctive use of L‐PRF in apical surgery may provide significant clinical benefits.

In recent years, patient‐related outcome measures (PROMs) have been increasingly recognized as essential in evaluating treatment success (Ibrahim and Dimick 2018; Neelakantan et al. 2022). Given that surgeries can be physically debilitating, it is valuable to assess patients' postoperative pain, swelling, and overall QoL. This case series observed a trend of no to mild pain at 24 h post‐apical surgery. While most patients experienced postoperative swelling, it was well controlled and did not require antibiotics. These findings align with previous studies reporting that the incorporation of autologous platelet concentrates in apical surgery enhances early postoperative QoL (Del Fabbro et al. 2012; Angerame et al. 2015). Additionally, systematic reviews have suggested that using autologous platelet concentrates in apical surgery offers a cost‐effective means to improve patients' QoL (Meschi et al. 2016; Del Fabbro et al. 2016). However, a more recent randomized controlled trial found no significant difference in QoL between patients treated with L‐PRF and those in the control group, despite the L‐PRF group reporting slightly lower pain levels during the first seven postoperative days (Meschi et al. 2018). Regarding postoperative swelling, Meschi et al. (2018) reported a high incidence of swelling within the first week after surgery, a finding consistent with this case series. The increased postoperative swelling associated with L‐PRF application may be attributed to the inflammatory response triggered by the abundant growth factors and cytokines released by L‐PRF. These biological mediators amplify the early inflammatory phase, increase vascular permeability, and contribute to transient postoperative swelling. Nevertheless, unlike swelling caused by infection, L‐PRF‐induced swelling is a controlled, physiological response that plays a role in the healing process.

## 5 Limitations

As this study is a case series, it lacks a control group to compare the outcomes of endodontic microsurgery with and without L‐PRF. Additionally, the follow‐up assessment was conducted at 6 months (with one case at 4 months) using only postoperative periapical radiographs. Given that 12 out of 13 cases had preoperative CBCT, it would be more appropriate to perform postoperative CBCT at the 1‐year review to ensure a more accurate evaluation of treatment outcomes, considering its superior sensitivity, specificity, and accuracy (Patel et al. 2009). Furthermore, pain and swelling assessments were not based on validated tools and were conducted via phone interviews without clinical evaluation, making the outcomes highly subjective. Other potential factors influencing healing, such as smoking status and underlying medical conditions, should also be considered in future studies to provide a more comprehensive understanding of treatment success.

Key questions remain unanswered regarding L‐PRF, which are essential for developing a standardized protocol for its use:

1. Should L‐PRF be applied as a standalone membrane, or should it be combined with cell‐occlusive membranes?
2. Should L‐PRF be used in conjunction with bone grafting materials?

## 6 Conclusion

The use of L‐PRF in endodontic microsurgeries remains to be assessed. Nonetheless, the favorable clinical and patient‐centered outcomes support its use. However, high‐quality studies, especially randomized controlled trials, are still required to evaluate the risks and benefits of using L‐PRFs in endodontic microsurgeries.

## Reporting Guidelines

Preferred Reporting Items for Case Reports in Endodontics (PRICE) 2020 Guidelines.

## Author Contributions

**Natrah Ahmad Fuad:** conceptualization, visualization, project administration, manuscript write‐up (original draft, review and editing). **Panagiotis Pitros:** visualization, project administration, manuscript review and editing. **Graeme Brown:** conceptualization, visualization, project administration. **Eleni Besi:** conceptualization, visualization, project administration, manuscript review.

## Ethics Statement

Approval was obtained from the Quality Improvement Project Assessment Team (QIPAT) at NHS Lothian. The document is present in the supporting document section.

## Consent

Informed, valid consent was obtained for clinical examination and further investigation from each patient. Consent documents are available upon request.

## Conflicts of Interest

The authors declare no conflicts of interest.

## Supporting information

LPRF PRICE‐2020‐Checklist.

## Data Availability

Data sharing is not applicable to this article as no new data were created or analyzed in this study.

## Appendix 1

**Root canal treatment/retreatment protocol**

- Local anesthetic (4% Articaine with 1:100,000/2% Lidocaine with 1:100,000 Adrenaline/3% Mepivacaine Plain).
- Rubber dam isolation.
- Surgical microscope (Global G6 series dental microscope).
- Creation of a glide path with size 10 K‐Flexofile.
- Canal preparation with ProTaper Next or ProTaper Gold rotary files (Dentsply Sirona).
- Gutta percha removal with ProTaper Next/ProTaper retreatment files (D series Dentsply)/Hedstroem files with or without the aid of solvent (eucalyptus oil).
- VDW Silver rotary machine (VDW.Silver).
- Working length determination with apex locator (JMorita Dentaport Root ZX) with or without intraoperative working length periapical radiograph.
- Irrigation with 5.25% sodium hypochlorite (CHLORAX 5.25% Cerkamed) and 17% EDTA (Endo‐Solution 17% Cerkamed). Final irrigation with 5.25% sodium hypochlorite.
- Irrigant activated via manual dynamic activation.
- Calcium hydroxide (Clacipast pH 12.5, Cerkamed) as intracanal medication for multiple‐visit RCT.
- Obturation with warm vertical condensation technique and zinc oxide eugenol sealer (Kerr Sealer).
- Light‐cured glass ionomer cement as provisional restoration and cotton pellet over the orifice for multiple‐visit RCT.
- SDR (Dentsply Sirona UK) and composite restoration for post‐obturation.
- Post‐obturation periapical radiograph.

## Appendix 2

**L‐PRF Protocol**

## Appendix 3 PRICE 2020 Flowchart

*From: Nagendrababu V, Chong BS, McCabe P, Shah PK, Priya E, Jayaraman J, Pulikkotil SJ, Setzer FC, Sunde PT, Dummer PMH (2020) PRICE 2020 Guidelines for reporting case reports in Endodontics: A consensus‐based development. *International Endodontic Journal* https://doi.org/10.1111/iej.13285.

For further details, visit: http://pride-endodonticguidelines.org/price/.
